# Cryo-EM structures of Kv1.2 potassium channels, conducting and non-conducting

**DOI:** 10.1101/2023.06.02.543446

**Published:** 2023-06-03

**Authors:** Yangyu Wu, Yangyang Yan, Youshan Yang, Shumin Bian, Alberto Rivetta, Ken Allen, Fred J. Sigworth

**Affiliations:** Department of Cellular and Molecular Physiology, Yale University School of Medicine, New Haven, Connecticut USA

## Abstract

We present near-atomic-resolution cryo-EM structures of the mammalian voltage-gated potassium channel Kv1.2 in open, C-type inactivated, toxin-blocked and sodium-bound states at 3.2 Å, 2.5 Å, 2.8 Å, and 2.9Å. These structures, all obtained at nominally zero membrane potential in detergent micelles, reveal distinct ion-occupancy patterns in the selectivity filter. The first two structures are very similar to those reported in the related Shaker channel and the much-studied Kv1.2–2.1 chimeric channel. On the other hand, two new structures show unexpected patterns of ion occupancy. First, in the toxin-blocked channel α-Dendrotoxin, like Charybdotoxin, is seen to attach to the negatively-charged channel outer mouth, and a lysine residue penetrates into the selectivity filter. Penetration by α-Dendrotoxin is however deeper than with Charybdotoxin, occupying two of the four ion-binding sites. Second, a structure of Kv1.2 in Na^+^ solution does not show collapse of the selectivity filter that was observed under similar conditions in the KcsA channel, but instead shows an intact selectivity filter with ion density in each binding site. We also attempted to image the Kv1.2 W366F channel in Na^+^ solution, but the protein conformation was seen to be highly variable and only a low-resolution structure could be obtained. These findings present new insights into the stability of the selectivity filter and the mechanism of toxin block of this intensively studied, voltage-gated potassium channel.

## Introduction

Among the most-studied ion channel proteins is the Kv1 family of tetrameric, six-transmembrane-segment, voltage-gated potassium (Kv) channels. The best-known member of this family is the *Drosophila* Shaker channel, first cloned in the late 1980s (Iverson et al., 1988; Papazian et al., 1987) and which quickly became the favorite object for biophysical studies due to its small size and ease of heterologous expression. The potassium current in the squid giant axon, first described by Hodgkin and Huxley (Hodgkin & Huxley, 1952) is carried by a closely related Shaker homologue (Rosenthal & Bezanilla, 2002).

In 2007 Long et al. (Long et al., 2007) published the 2.4Å X-ray structure of a rat Shaker-family channel, the “paddle chimera” Kv1.2 in which a portion of the voltage-sensor domain was replaced by the corresponding Kv2.1 sequence. This structure has been a watershed in the structural understanding of many functional measurements and has formed the basis of computational studies of voltage-sensing and gating mechanisms. A subsequent cryo-EM structure of the same channels reconstituted into nanodiscs (Matthies et al., 2018) has excellent agreement with the X-ray structure. Meanwhile, a very welcome recent development is the cryo-EM structure of the original Shaker channel itself (Tan et al., 2022).

The high-resolution structure of the Kv1.2 paddle chimera channel has been the basis for much research, but the native Kv1.2 channel has been more used for functional studies (Ishida et al., 2015; Suarez-Delgado et al., 2020; Wu et al., 2022). The properties of the native and chimeric channels are similar but not identical (Tao & MacKinnon, 2008). One goal of the current study was to examine the structure of the native Kv1.2 channel.

Inactivation is an important process that diminishes voltage-gated channel current even when the activating membrane depolarization is maintained. A relatively slow inactivation process, usually called C-type inactivation (Hoshi et al., 1991) involves conformational changes in the pore domain and the selectivity filter. Various mutations in the pore domain have been shown to accelerate or impede C-type inactivation. The Shaker pore-domain mutation W434F renders the channel almost completely non-conductive through a process like C-type inactivation (Perozo et al., 1993; Pless et al., 2013; Suarez-Delgado et al., 2020; Yang et al., 1997). Structures of this mutant (Tan et al., 2022) and of a corresponding mutant Kv1.2–2.1 paddle chimera channel (Reddi et al., 2022) show a dramatic dilation of the selectivity filter that disrupts two of the four ion-binding sites in the selectivity filter. We report here the structure of the corresponding W366F mutant in the native Kv1.2 channel background, which shows a very similar dilation.

An important aspect of C-type inactivation is that it is enhanced when potassium ions are absent. A structural study of the bacterial KcsA channel (Zhou et al., 2001) and a molecular-dynamics simulation of the Kv1.2 paddle chimera (Jensen et al., 2012) show very dramatic but opposing effects when the potassium ion concentration is reduced. In KcsA the replacement of most K^+^ with Na^+^ results in a collapse of the selectivity filter region, occluding the two central ion-binding sites. On the other hand, a lengthy molecular-dynamics simulation of deactivation in the Kv1.2–2.1 chimera channel shows that, with the closing of the channel gate, there is a complete loss of water and ions from the cavity below the selectivity filter. Under these conditions however the selectivity filter remains intact and is populated by K^+^ ions (M. Ø. Jensen, personal communication). In our current study we sought to observe experimentally the structure of the Kv1.2 channel when potassium ions are absent.

Finally, Kv channels are targets for many toxins that inhibit channel conductance, with a common mechanism being direct occlusion of the pore. This occlusion has been seen directly in one case, in crystal structures of the Kv1.2 paddle-chimera channel with charybdotoxin (CTx) bound (Banerjee et al., 2013). Dendrotoxins (DTxs) produced by the mamba snake *Delodiaspis* are distinct from CTx but also block Kv channels with high potency and selectivity (Gasparini et al., 1998). In this study we have determined the structure of Kv1.2 with α-DTX bound.

## Results

### Overview of mammalian Kv1.2 structure

For this study we employed a full-length Kv1.2 α-subunit construct containing three mutations in disordered regions of the N-terminus and S1-S2 linker. We also used a β_2_-subunit having five mutations chosen to neutralize positive charges on the cytoplasmic face; this removes the strong interactions with cryoEM substrates that were observed by Matthies et al. (Matthies et al., 2018) with no effects on secondary structure. As our interest is with the transmembrane portion of the alpha subunit, for the purposes of this paper we term the resulting α_4_β_4_ complex the native Kv1.2 channel.

**Figure 1,:**
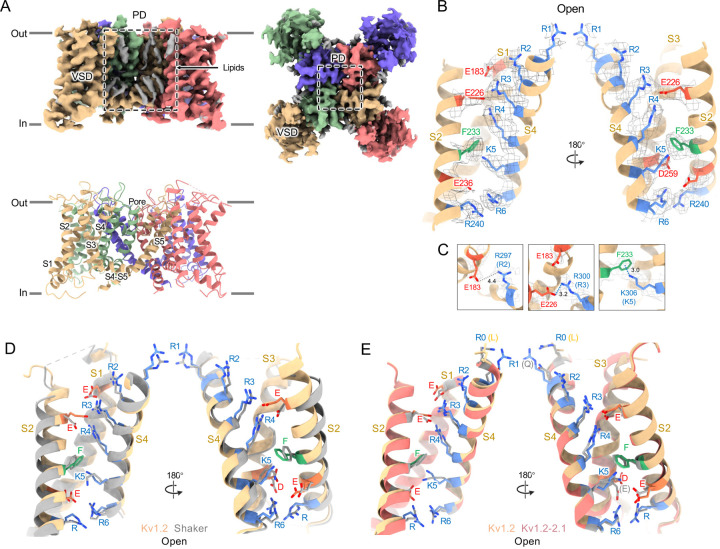
Open Kv1.2 overall structure. (A) Side and top view of the native Kv1.2 cryo-EM density map (upper panel) and model (lower panel). Lipid densities are colored gray in the map. (B) Side view of VSD structure and map density of Kv1.2 (yellow). (C) The relative positions of the interacting residues R2 and E138 (upper), R3 and E226 (middle), K5 and F233 (lower) are shown. (D) Superposition showing the very close match of Kv1.2 (yellow) and Shaker (gray) VSD structures. (E) Superposition of Kv1.2 WT (yellow) and Kv1.2–2.1 (pink) VSD structures. Positively charged, negatively charged and aromatic residues are shown as blue, red and green, respectively. VSD, voltage-sensing domain; PD, Pore domain.

The constructs encoding the Kv1.2 native or W366F mutant α-subunits, along with the β_2_ subunits, were expressed in *Pichia pastoris* essentially as described (Long et al., 2005). Channel complexes were affinity-purified in the presence of dodecylmaltoside detergent and subjected to size-exclusion chromatography in buffers containing either 150 mM K^+^ or 150 mM Na^+^ ions. The channel complexes were plunge-frozen on grids for cryo-EM analysis. Focusing on the transmembrane region of the Kv1.2 channel complexes, we obtained structures of nominally open, C-type inactivated, DTX-bound, and K^+^ free states at resolutions of 3.2 Å, 2.5 Å, 2.8 Å and 2.9 Å respectively. Most main-chain and sidechain densities were clearly visible in the resulting maps allowing atomic models to be built with high confidence. Structures of the native Kv1.2 channel (Figure 1A–C, and Figure 1—figure supplement 1) are very similar to those of the Kv1.2–2.1 chimera (Long et al., 2007) and *Drosophila* Shaker (Tan et al., 2022) channels. As observed in other Kv1 and Kv2 structures, the voltage-sensing domains (VSDs) contain the membrane-spanning S1-S4 helixes (Figure 1D and E) while the S5, S6, pore helix and selectivity filter P-loop form the ion-conduction pore in a domain-swapped configuration. Densities of lipid attached to the VSDs and pore domains (PDs) are clearly visible in the Kv1.2 maps obtained in this study (Figure 1A).

**Figure. 2:**
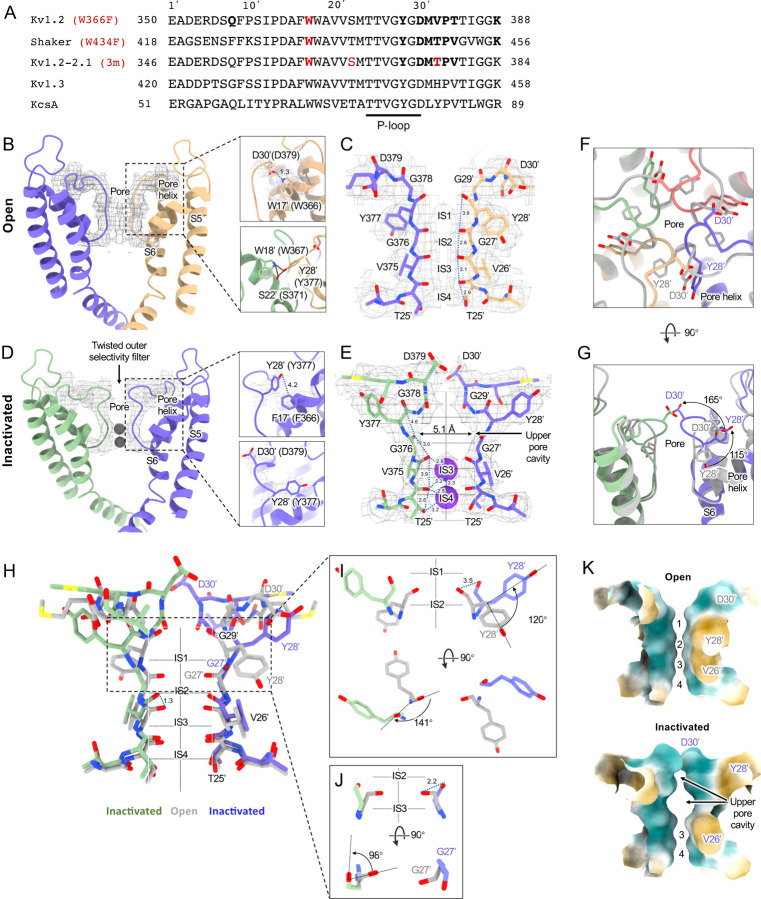
Kv1.2 pore domain and selectivity filter structures in inactivated and open conformation. (A) Sequence alignment of potassium channels in the S5-S6 linker region, with the relative numbering by Miller (1990) indicated at top. (B) Side view of opposing subunits in the open Kv1.2 pore domain. Relative positions of Y28’, W17’ and D30’ are shown in the right-hand panels. Shown with a dashed blue line is the key hydrogen bond between D30’ and W17’ that is eliminated in the W17’F (W366F) mutant. (C) Side view of opposing Kv1.2 P-loops. On the left is shown the Kv1.2 residue numbering, and on the right the Miller numbering. (D) Side view of the inactivated Kv1.2 W17’F pore domain. The new locations of Y28’, F17’ and D30’ are shown in the right panels. (E) Side view of the Kv1.2 P-loop in the inactivated conformation. In the large upper-pore cavity the G27’ and G29’ carbonyls are 5.1 and ** 11? ** Å apart. Potassium ions are shown as purple balls. (F) Top view of the Kv1.2 outer pore in inactivated (colored) and open (grey) states. In the inactivated channel the displaced Y28’ ring of one subunit is in the position occupied by D30’ - in the neighboring subunit - of the open channel. (G) The corresponding side view. The large rotation of the Y28’ side chain and flipping of D30’ are indicated by curved arrows. (H) Side view of the P-loop in inactivated (colored) and open (grey) conformations. (I,J) Details of Y28’ G27’ carbonyl and side chain reorientation from open (grey) to inactivated (colored) states are shown in side view (upper) and top view. (K) Surface renderings of open and inactivated selectivity filter region. Hydrophilic and hydrophobic surfaces are shown in teal and cantaloupe, respectively.

As in the other homologues an open S6 gate is visible in the detergent-solubilized Kv1.2 structure; this is expected for the open conformation at zero membrane potential. Seeing no evidence of a barrier to ion flux, we call this the open-channel structure (Figure 2A–B).

In the critical region of the voltage-sensor domain (VSD) the sidechains of the voltage-sensing Arg and the coordinating Glu and Asp residues, as well as the charge-transfer center phenylalanine, are essentially superimposable with Shaker (Figure 1D), with an RMS difference of all Arg sidechain atoms of 0.85 Å. As Kv1.2 is a member of the Shaker potassium channel family and has 68% amino-acid identity with *Drosophila* Shaker, it is not surprising that the fold is very similar.

Despite the very closely matched open-state structures of the VSDs it is therefore puzzling that the total gating charge movement in Shaker channels is larger, about 13 elementary charges per channel, while it is only 10 e_0_/channel in Kv1.2 (Ishida et al., 2015). The most likely explanation is that in Kv1.2 there is some additional restraint, lacking in Shaker, on the physical displacement of each S4 helix at negative membrane potentials.

In the paddle chimera a region including the N-terminal half of the S4 helix in Kv1.2 is replaced by that of Kv2.1. One difference in the voltage-sensing residues is that a glutamine replaces the arginine at the first position (R1) in the chimera. Another difference is that there is an earlier Arg residue R0 that replaces leucine, but is unlikely to contribute to charge movement. A direct measurement of charge movement in the paddle chimera is lacking, but macroscopic activation and inactivation show slightly lower voltage sensitivity of (Tao & MacKinnon, 2008) as if its gating charge movement were reduced. Nevertheless the structures of the S4 regions are very similar: comparing Kv1.2 with the paddle chimera, the rms deviation of Arg sidechain atoms is less than 0.75 Å.

### Channel inactivation

Figure 2 compares the structure of the open channel with that of the Kv1.2 W366F mutant. While the corresponding mutation W434F in Shaker essentially abolishes ion current through the channel (Yang et al., 1997) the Kv1.2 mutant allows currents to flow transiently on depolarization, but decay to less than 3% of the peak in 80 ms (Figure 2—figure supplement 1). Inactivation in these channels can be slowed by raising extracellular K^+^ or by applying the tetraethylammonium ion (Suarez-Delgado et al., 2020), properties that are hallmarks of C-type inactivation. Here we shall call the cryo-EM structure of Kv1.2 W366F the “inactivated channel” structure (Figure 2—figure supplement 2), keeping in mind that other distinct inactivated channel conformations are likely to exist.

For comparison with structures of other voltage-gated potassium channels, we borrow from Miller (1990) the numbering of the 39 residues in the S5-S6 linking region, which we will denote as residue numbers 1’ to 39’. (Figure 2A). The 39-residue numbering is appropriate for Kv1 through Kv4 channels (Shealy et al., 2003) and is convenient in comparisons with KcsA, because its residue numbering differs by exactly 50. In the Miller system for example the Kv1.2 W366 residue is W17’, and we will denote the W366F mutant channel Kv1.2-W17’F.

The selectivity filter of potassium channels consists of an array of four copies of the extended loop (the P-loop) formed by a highly conserved sequence, in this case TTVGYGD. The main-chain carbonyl oxygens of the residues TVGY, positions 25’ to 28’, delineate ion binding sites S3, S2 and S1, respectively, while the lower (most intracellular) edge of binding-site S4 is formed by the T25’ hydroxyl. Two residues anchor the outer half of the selectivity filter and are particularly important in inactivation mechanisms (Figure 2B, right panels). Normally, the tyrosine Y28’ (Y377 in Kv1.2) is constrained by hydrogen bonds to residues in the pore helix and helix S6 and is key to the conformation of the selectivity filter. The final aspartate of the P-loop, D30’ (D379 in Kv1.2) is normally located near the extracellular surface and has a side chain that also participates in H-bonds with W17’ (W366 in Kv1.2) on the pore helix.

The difference between the open and inactivated Kv1.2 structures, like the difference in Kv1.2–2.1 (Reddi et al., 2022) and Shaker (Tan et al., 2022) can be imagined as resulting from a two-step process. The first step is a partial twist of the P-loop backbone involving D30’. When W17’ is mutated to phenylalanine, it is no longer an H-bond donor (Figure 2D right upper panel). The resulting destabilization of D30’ allows it to reorient toward the external water-filled vestibule.

The second step is the reorientation of Y28’ and further twisting of the polypeptide backbone. Y28’ normally participates in H-bonds with the pore-helix residues W18’ and S22’ (Figure 2D right lower panel), but with the release of D30’, and presumably the entry of water molecules into the space surrounding the P-loop, the side chain of Y28’ also reorients toward the external solution, filling some of the original volume occupied by the side chain of D30’. The reorientation of the phenol group of Y28’ is through a very large pitch angle of about 120° (Figure 2G,H,I). The reorientations of the D30’ and Y28’ side chains drag and twist the backbone. The result is an enlargement of the ion-binding site IS2 to a width of 5.1Å (Figure 2E) and an even wider (11 Å) cavity is formed at the level of IS1. There are no clear ion densities in the upper selectivity filter.

Meanwhile, the lower binding sites IS3 and IS4 show relatively small disturbances in the inactivated state. The largest change is a rise of 1.3Å in the position of the G27’ carbonyl, the one which defines the top of IS3 (Figure 2J). As in the inactivated Shaker structure (Tan et al., 2022) strong ion densities are seen in IS3 and IS4. Molecular dynamics simulations by Tan et al. based on the Shaker-W17’F structure show that IS3 and IS4 are simultaneously occupied by K^+^ ions in the inactivated state. It appears that the lack of Ion conduction is caused by the immobility of these bound ions: they can be displaced, although rarely, if a very high membrane potential (450 mV) is applied.

Might symmetry-breaking accompany Kv1.2 inactivation? MD simulations of inactivated paddle chimera, KcsA and hERG channels (Kondo et al., 2018; Li, Shen, Reddy, et al., 2021; Li, Shen, Rohaim, et al., 2021), all homotetramers, show a change to two-fold symmetry in the pore region such that the selectivity filter has distinct conformations for each pair of opposing subunits. However, we were not able to detect any evidence of reduced symmetry in the inactivated Kv1.2 channel. We were able to build the atomic model unambiguously into the 2.5 Å map that was obtained with C4 symmetry imposed. Further, there was no evidence of broken symmetry even when using symmetry expansion and local refinements of the cryo-EM dataset, for example as employed by Zhang et al. (Zhang et al., 2021).

Functional interactions have been observed between residues in the voltage-sensing domains and pore residues involved in C-type inactivation (Bassetto et al., 2021; Conti et al., 2016). A comparison of our open-channel and inactivated Kv1.2 structures show subtle but noticeable differences in the VSDs. Salt bridges involving the S4 Arg and Lys residues are shifted slightly (Figure 2—figure supplement 3A–D). Arg300 (R3) is in close proximity to Glu226 on the S2 helix for the open channel, while R3 is closer to Glu183 in the S2 helix. The Glu226 side chain adopts a visible interaction with R4 in the inactivated state. In both open and inactivated states of Kv1.2, K5 interacts more closely with S2 Phe233 than is the case in the paddle chimera channel (Figure 1E).

While the VSD helices in Kv1.2 and the inactivated Kv1.2-W17’F superimpose very well at the top, there is a general twist of the helix bundle that yields an overall rotation of about 3° at the bottom of the VSD. In moving to the inactivated state the axes of the helices S0, S1 and S2 tilt by 6°, 4° and 3.2° in a clockwise direction as viewed from the cytoplasmic side, while S4 is stationary. This lower VSD rotation provides a good explanation of the shifts in the S2 residues Glu183 and Glu226 that interact with R2 and R3 (Figure 2—figure supplement 3E–F). In contrast to Kv1.2, comparisons of both Shaker and Kv1.2–2.1 structures reveal almost complete superposition of the VSD domains between open and inactivated states (Figure 2—figure supplement 3G–H).

### A dendrotoxin lysine blocks Kv1.2 by occluding two ion binding sites.

**Figure. 3:**
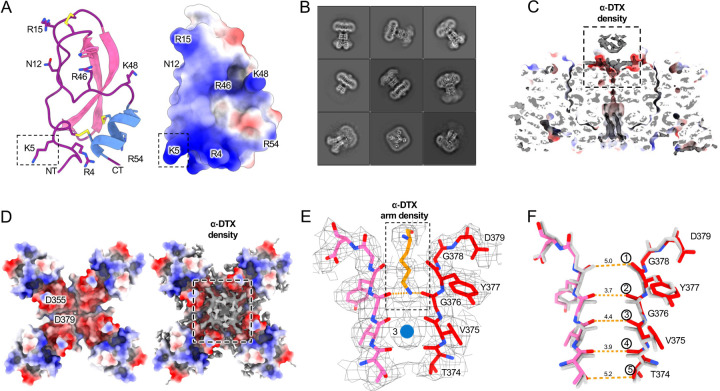
Kv1.2 DTX bound structure. (A) DTX model and electrostatic surface view. Positively-charged sidechains are shown, and the three disulfide bridges are shown in yellow. (B) Representative 2D classes of the DTX-bound Kv1.2 particles. (C) The Kv1.2-DTX structure is shown as electrostatic surface view with DTX cryo-EM density (gray) visible at top. (D) Top view of the Kv1.2 DTX structure is shown as electrostatic surface view with (right panel) and without (left panel) DTX cryo-EM density (gray). (E) Side view of the Kv1.2-DTX selectivity filter. Ion density is marked as a blue ball. (F) Superposition of Kv1.2 DTX (colored) and Kv1.2 WT (gray) selectivity filter structures. Orange dashed lines show the distances between carbonyl groups of the Kv1.2 DTX structure. The corresponding distances in the absence of toxin are 5.0, 4.7, 4.7,4.7 and 5.5Å.

Dendrotoxins are peptide neurotoxins from mamba snakes that bind with nanomolar affinities and block potassium channels. Alpha-dendrotoxin (α-DTX) consists of a peptide chain of 59 amino acids stabilized by three disulfide bridges (Figure. 3A) and, like other dendrotoxins, exhibits the same fold as Kunitz protease inhibitors (Skarzynski, 1992). Arginine and lysine residues are concentrated near the N-terminus (Arg3, Arg4, Lys5), the C-terminus (Arg54, Arg55) and at the narrow β-turn region (Lys28, Lys29, Lys30). The conserved lysine residue near their N-terminus (Lys5 in alpha-DTX) is essential for biological activity (Harvey et al., 1997). Its sidechain protrudes from the surface of the molecule and is a likely candidate for strong interaction with the channel pore.

The IC_50_ for block of Kv1.2 by α-DTX is estimated as 0.4 to 4.0 nM in oocytes and 2.8 to 17 nM in mammalian cells (Harvey, 2001). By adding 100 nM α-DTx to detergent-solubilized Kv1.2 protein we obtained a cryo-EM structure at 2.8 Å resolution of the complex (Figure 3 and Figure 3—figure supplement 1). The transmembrane domain shows an additional cap of density at the top of the channel pore (Figure 3B–D), as would be expected for binding of the positively-charged toxin with the extensive negative charges at the pore entrance. The toxin density was not interpretable, as would be expected for the overlapping density of alternative poses of the asymmetric ligand bound to the fourfold-symmetric channel. Zhang et al. (Zhang et al., 2021) recently showed that small asymmetric ligands can be distinguished in large single-particle datasets, but we were unable to obtain the requisite C1 reconstruction from our dataset.

In the selectivity filter of the toxin-bound channel (Figure 3E) a continuous density is seen to extend downward from the external site IS0 through to the boundary between IS1 and IS2. This density is well modeled by an extended Lys side chain from the bound toxin, with the terminal amine coordinated by the carbonyls of G27’. Below it, binding site IS2 has a low density, most likely occupied by a water molecule. Only binding site IS3 shows the strong density consistent with a bound ion. Binding site IS4 has low density, but at its lower boundary, coordinating the T25’ side chains, is a flat density that is likely to reflect water molecules bordering the selectivity filter entrance.

**Figure. 4:**
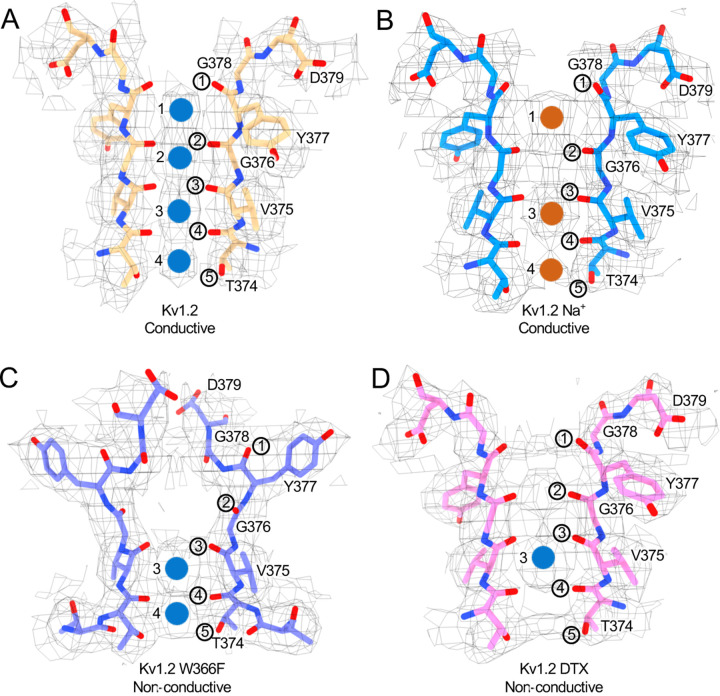
Summary of Kv1.2 conductive and non-conductive pores. Selectivity filter structures of (A) Kv1.2 WT, (B) Kv1.2 WT Na+ bound, (C) Kv1.2 W366F, and (D) Kv1.2 DTX bound. Potassium ions and sodium ions are shown as blue and orange balls, respectively. Circled numbers label the P-loop carbonyl oxygens.

Toxin binding shrinks the distances between opposing carbonyl oxygens in the selectivity filter, forming a narrower tunnel into which the Lys side chain fits (Figure 3F). The second and fourth carbonyl oxygen distances are substantially reduced from 4.7 Å and 4.6 Å in open state to 3.7 Å and 3.9 Å, respectively (Figure 4E). In a superposition of Kv1.2 open-state and α-DTX-bound P-loop structures there is also an upward shift of the first three carbonyl groups by 0.7~1.0 Å (Figure 4F).

We also tried mixing 100nM α-DTX with the Kv1.2-W17’F protein sample in an attempt to observe a pore-blocker-bound inactivated structure. However, we found no evidence of bound toxin density in the cryo-EM 2D classes. This is to be expected from the large rearrangement of the extracellular pore entrance in the mutant, which abolishes the IS1 site and reorients the carbonyls of G376 (Figure 3F), eliminating all of the interactions with the Lys sidechain of the toxin.

Through an elegant series of crystal structures, Banerjee et al. (Banerjee et al., 2013) were able to obtain an unambiguous structure of the scorpion toxin charybdotoxin (CTX) bound to the Kv1.2–2.1 paddle chimera. CTX and α-DTX are unrelated toxins, having different folds and different sizes (4 and 7 kDa). We find that the mechanism of pore blockage is also distinct. The key pore-blocking residue in CTX, K27, penetrates into the selectivity filter and perturbs binding site IS1 by binding to the outermost carbonyls (those of Y377). On the other hand, the terminal amine of a lysine in α-DTX is deeply wedged at the second set of carbonyls, narrowing both IS1 and IS2 while displacing ions from the sites (Figure 3—figure supplement 2A). CTX does not cause narrowing of the selectivity filter or displacements of the carbonyls (Figure 3—figure supplement 2B).

### The Kv1.2 selectivity filter in K^+^-free medium

The X-ray crystal structure of the KcsA channel in low K^+^ solution (Zhou et al., 2001) shows a collapsed selectivity filter, in which the residues V26’ and G27’ are rearranged, abolishing the binding sites IS2 and IS3. On the other hand, Shaker channels are able to conduct Na^+^ in the absence of K^+^ (Melishchuk et al., 1998). We obtained the structure of Kv1.2 in a zero K^+^ solution, with all potassium replaced with sodium, and were surprised to find that it is little changed from the K^+^ bound structure (Figure 4B and Figure 4—figure supplement 1 and 2). Ion densities are seen in the IS1, IS3 and IS4 ion binding sites, but the selectivity filter shows a general narrowing as would be expected for binding of sodium ions. The second, third and fourth carbonyl oxygen distances are reduced from 4.7 Å, 4.7 Å and 4.6 Å in the open state to 4.4 Å, 3.9 Å and 4.5 Å, respectively. The rest of the channel structure is very little perturbed.

### Structure of the W366F mutant in K^+^-free medium

**Figure. 5:**
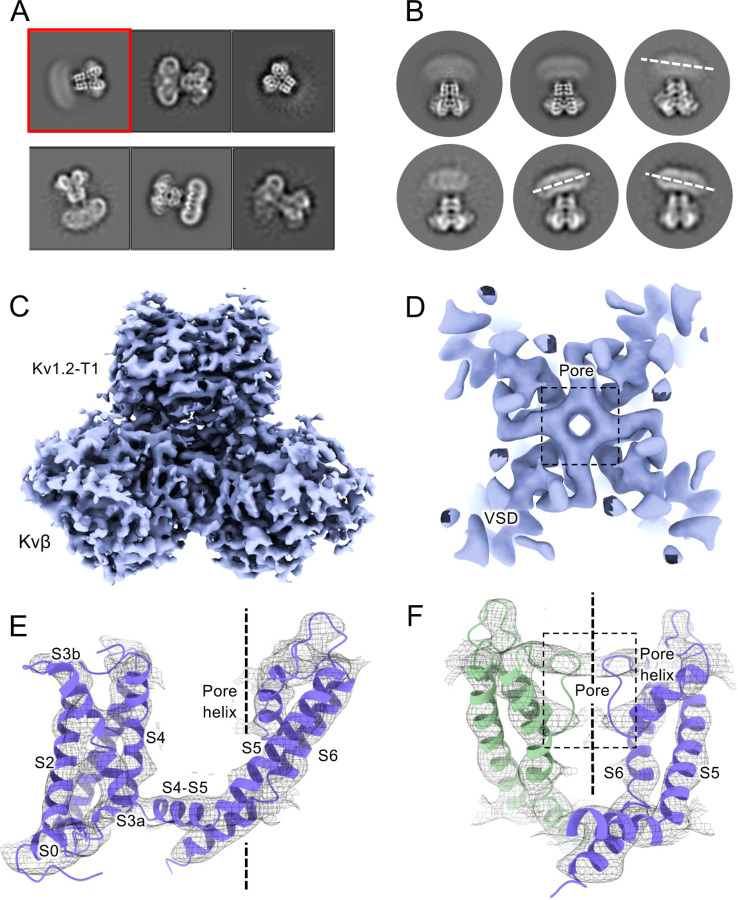
Cryo-EM analysis of Kv1.2 W366F in Na^+^. (A) Representative 2D classes. (B) Second round of classification of first class in (A), outlined in red. Wobbling of the transmembrane domain is illustrated by the white dashed lines. (C) Density of intracellular domain, obtained by focused refinement. (D) Top view of TMD. (E) Overall TMD map density fits with Kv1.2 W366F model. (F) Pore domain density fitting shows the absence of pore-loop density.

We also collected cryo-EM data from the W366F mutant channels with Na^+^ replacing K^+^ in the final size-exclusion chromatography step. In Shaker the corresponding W434F mutant channel stably carries large Na^+^ and Li^+^ currents in the absence of K^+^ (Starkus et al., 1998). In 2D classification of the single-particle images we saw large variability in the structure, as if the connection between the transmembrane domain and the intracellular domains (the T1 domain and the Kvβ_2_ subunits) was highly flexible (Figure 5A–B). The intracellular domains were resolved to 3.0 Å resolution in a focused reconstruction (Figure 5C) but the complementary focused reconstruction of the transmembrane domain yielded only about 7 Å resolution. Nevertheless this low-resolution map matches the secondary structure of the Kv1.2 W366F mutant in K^+^ solution (Figure 5D), with the possible exception of low density in the selectivity filter region (Figure 5E–F). We conclude that the structure of this mutant channel becomes unstable in the absence of K^+^, as if the tight binding of K^+^ ions is required to stabilize the altered selectivity filter.

## Discussion

The potassium channel signature sequence TTVGYGD was first identified by Heginbotham et al. (Heginbotham et al., 1994) as critical for potassium selectivity. The residues fold as an extended loop (the P-loop) and is part of the P-region, about 20 residues of conserved sequence located between the S5 and S6 helices. We use here a numbering system of the entire linking region between S5 and S6 identified in Shaker by Miller (Miller, 1990). The P-loop (residues 24’–30’ in this numbering, TTVGYGD) produces four ion binding sites (Doyle et al., 1998) through which K^+^ ions pass sequentially in a knock-on fashion. The geometry and flexibility is finely tuned to allow high K^+^ selectivity with a high transport rate (Noskov & Roux, 2006; Roux, 2005).

In this paper we have considered the ion occupancy and conformation of the P-region under four conditions: the conducting state, a C-type inactivated state, block by dendrotoxin, and the absence of permeant ions. In the conducting state, our cryo-EM structure of native rat Kv1.2 in detergent is very similar to the structure of *Drosophila* Shaker in lipid nanodiscs (Tan et al., 2022). Further, apart from local differences from the chimeric region of the voltage sensor domains (VSDs), we find that our structure is essentially identical to the structures of the rat Kv1.2–2.1 paddle chimera as obtained from X-ray crystallography in the presence of lipid and detergent (Long et al., 2007), and from cryo-EM imaging of the paddle chimera in lipid nanodiscs (Matthies et al., 2018). The conformation of the entire transmembrane region of the Kv1.2 channel and its variants appears to be remarkably insensitive to its environment, whether lipid or detergent.

### Inactivation

After the opening of the intracellular activation gate formed by the S6 helices, C-type inactivation (Hoshi et al., 1991) causes channel currents spontaneously to switch off. The inactivation process is very sensitive to mutations in the selectivity filter and nearby residues of the P-region and is influenced by the state of the S6 gate (Cuello et al., 2017; Li, Shen, Rohaim, et al., 2021) and the VSDs (Bassetto et al., 2021; Conti et al., 2016). Low K+ concentrations, especially in the extracellular solution, accelerate inactivation (Levy & Deutsch, 1996).

In the KcsA channel the structural change upon inactivation was observed as a collapse of the selectivity filter in crystals soaked in low-K^+^ solution (Zhou et al., 2001). There is a pinching of the selectivity filter at the level of G27’ such that ions can no longer bind at sites IS2 and IS3, completely blocking the single-file ion permeation (Cuello et al., 2010; Li et al., 2018). Also, in an alternative mechanism (Rohaim et al., 2022) KcsA can become nonconductive through a general narrowing of the selectivity filter.

In the case of Kv1.2, structural and molecular-dynamics studies have focused on the mutation W17’F (W366F in Kv1.2) which induces an inactivation-like process that is slowed in the presence of extracellular K^+^ ions or pore blockers. In Shaker this mutation (W434F) causes the near total loss of open-channel current (Li et al., 2018; Perozo et al., 1993; Yang et al., 1997); in Kv1.2 it greatly accelerates inactivation (Suarez-Delgado et al., 2020).

In a very extensive review of experimental results in Shaker channels, Hoshi and Armstrong (Hoshi & Armstrong, 2013) pointed out that as a mechanism for inactivation, dilation of the external pore is more likely than constriction. Considering the Shaker W17’F phenotype as a model for inactivation, and noting that W17’ interacts with both D30’ and the central tyrosine Y28’ in the open state (Lueck et al., 2016; Pless et al., 2013), they predicted in homology modeling that inactivation induces a rotation of the Y28’ ring which in turn dilates the outermost binding site IS1 of the selectivity filter. They predicted a rotation of Y28’ also in the case of the inactivation-causing mutation T32’A. The predicted rearrangements of the Y28’ ring are small compared to the massive remodeling of the outer pore that we and others have now observed in structures of W17’F mutants, but nevertheless these authors were correct in predicting an expansion rather than collapse of the selectivity filter. Tan et al. (Tan et al., 2022) report an expansion of the outer pore of Shaker, as quantified as the distance between the Y28’ alpha-carbons, from 8.6 to 12.8 Å. The corresponding expansion in the Kv1.2 paddle chimera (Reddi et al., 2022) is from 7.2 to 10.7 Å; in Kv1.2 in this work it is from 7.4 to 10.8 Å.

A feature of the inactivated state structure reported here and in the other two W17’F structures (Reddi et al., 2022; Tan et al., 2022) is the high occupancy by potassium ions of the two remaining binding sites IS3 and IS4. During normal conduction, ion binding sites in the selectivity filter are usually occupied by K^+^ and water molecules in alternation. However, in molecular-dynamics simulations based on their Shaker W434F structure, Tan et al. (Tan et al., 2022) observed two tightly-bound, adjacent, K^+^ ions simultaneously present in IS3 and IS4. The ions did not leave the sites during a (relatively long) 2 µs simulation with 300mV membrane potential applied, but two knock-on permeation events were observed during 1µs at the very high potential of 450 mV. A similar result was obtained from MD simulations of the Kv1.2–2.1 chimera where the W17’F mutation was artificially introduced (Conti et al., 2016; Kondo et al., 2018). Those simulations showed small shifts in the geometry of IS3 and IS4 that, like those seen by Tan et al., produced simultaneous occupancy by K^+^ ions and blockage of ion permeation. A similar tight binding of adjacent K^+^ ions seems also to block K+ permeation in the structure of an inactivated Kv1.3 channel (Liu et al., 2021).

**Figure. 6:**
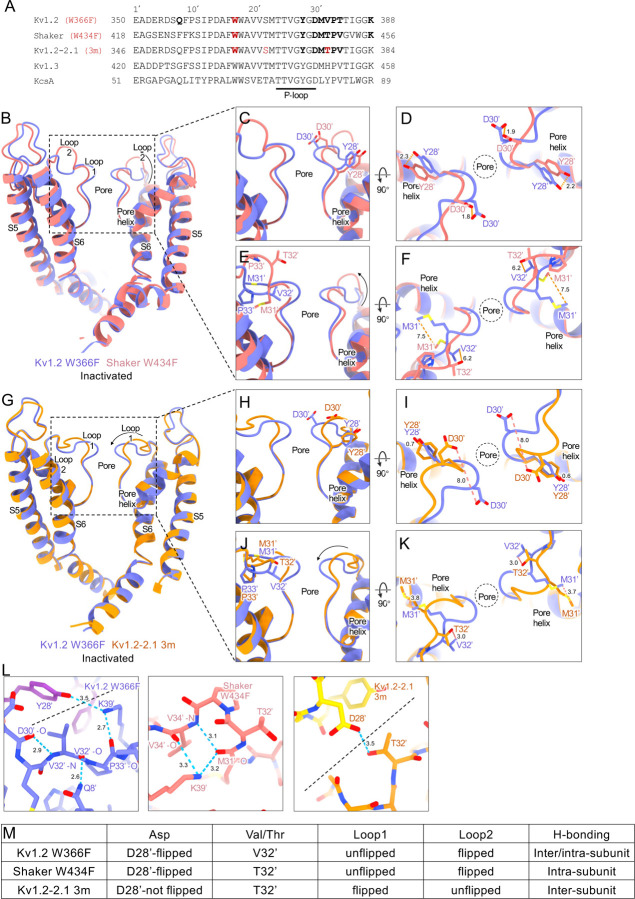
Structural comparison of inactivated Kv channels. (A) Sequence alignment of potassium channels under Miller’s numbering system. Side view structural superposition of Kv1.2 W366F and Shaker W434F (B), Kv1.2–2.1 3m (G) pore domains. Loop 1 conformational changes between Kv1.2 W366F and Shaker W434F side view (C), top view (D); Kv1.2–2.1 3m side view (H), top view (I). Loop 2 conformational changes between Kv1.2 W366F and Shaker W434F side view (E), top view (F); Kv1.2–2.1 3m side view (J), top view (K). (L) H-bonds among the inactivated Kv channels, adjacent subunits are shown as different color with a dashed line. (M) Table lists of the differences among the inactivated Kv channels.

The structures of the corresponding mutants Shaker-W17’F (Tan et al., 2022) and the Kv1.2–2.1–3m paddle chimera (which contains the mutations W17’F, S22’T and V32’T) (Reddi et al., 2022), show almost identically distorted selectivity filters (Fig. 6B, G) but with differences in the extracellular turret regions that are likely to affect the stability of the inactivated state. (Figure 6E, F, J, K). To describe the differences among all the inactivated channels, we refer to outer mouth loops 1 and 2 (Figure 6B, G). In each of the inactivated channels the Y28’ side chains move into the volume normally occupied by D30’. The D30’ residue is then located in the extracellular loop1 and in similar positions in Kv1.2 and Shaker W17’F (Fig. 6C,D). However, in Kv1.2–2.1–3m the flipped loop 1 places D30’ in a location 8Å from that in the other two channels (Figure 6H, I).

In the middle of loop 2, located in the outer turret, is the residue at the 32’ position, either Val or Thr (Figure 6E, F, J, K) which is seen in functional studies to have a strong influence on inactivation. The Kv1.2–2.1–3m construct has the V32’T mutation which would tend to stabilize the inactivated state by forming an H-bond to the flipped D30’ in the adjacent subunit (Figure 6L right panel). The corresponding residue in Shaker-W17’F is a Thr residue as well, but it does not participate in H-bonds due to the flipped loop 2. Instead, the flipped loop 2 is stabilized by K39’, located on the pore helix, forming H-bonds with the main-chain loop 2 of an adjacent subunit (Figure 6L middle panel). As for Kv1.2-W17’F, the corresponding residue is a unique V32’ that participates in an H-bond network that would tend to stabilize the inactivated conformation (Figure 6K, L left panel).

Similar but smaller reorientations of the residues D30’ and Y28’ are observed in the cryo-EM structure of the Kv1.3 mutant H20’N (H451N) (Liu et al., 2021), which also has greatly accelerated C-type inactivation. The mutation is at the position of S20’ in Kv1.2, which participates in an H-bond network with Y28’ in the open channel. The structure of the Kv1.3 mutant shows a distorted S2 site with no ion density, and an IS1 site that is dilated by 2–3 Å compared to the complete loss of IS1 and a dilation of about 7 Å in our Kv1.2 inactivated channel (Figure 6—figure supplement 1B,C).

**Figure. 7:**
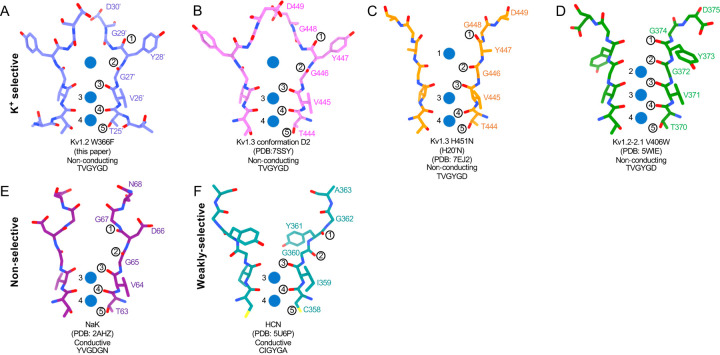
Structural comparison of Kv1.2 W366F with various channel pores. Selectivity filter structures and P-loop sequences of potassium selective non-conducting (A) Kv1.2 W366F in purple, (B) Kv1.3 alternate conformation in pink, (C) Kv1.3 H451N in orange, (D) Kv1.2–2.1 V406W in green. E,F Pore regions of less-selective channels, with corresponding pore sequences: the non-selective, conducting NaK in dark purple (E) and the weakly-selective, conducting HCN in light green (F). Potassium ions are shown as blue balls. Circled numbers enumerate the pore-forming carbonyl oxygens. Carbonyl (2) faces the adjacent subunit in the clockwise direction in all but the HCN channel in panel F.

Are the structures of the various mutant channels valid models for C-type inactivation? In a recent cryo-EM study (Selvakumar et al., 2022) the human Kv1.3 channel was found spontaneously to exist in alternative conformations D1 and D2 that appear to be inactivated states. The D1 structure shows a large displacement of D30’ and is reminiscent of the cryo-EM structure of the Kv1.3 rapidly-inactivating mutant H20’N (Liu et al., 2021). It exhibits a partially twisted P-loop (Figure 7C, Figure 2—figure supplement 2 and Figure 7—figure supplement 2B) as if it were an intermediate state between open and the W17’F structures. Meanwhile the D2 structure is very similar to that of the W17’F variants considered here, strongly supporting the widely-held view that these mutant channels are in a conformation close to, if not identical to, a true C-type inactivated state.

The large expansion of the outer selectivity filter in W17’F channels is reminiscent of the large extracellular vestibules observed in NaK and HCN channels (Lee & MacKinnon, 2017; Shi et al., 2006) which are weakly ion-selective (Gauss et al., 1998; Ludwig et al., 1998; Santoro et al., 1998). In HCN the residue at position 22’ (Thr in Shaker, Ser in Kv1.2) is replaced by Cys, but this change does not explain reduced selectivity (Zhou & MacKinnon, 2004). The reduced selectivity is likely arises from the existence of only two ion-binding sites. The IS3 and IS4 sites are formed in the same way as in the Kv channels (two carbonyl oxygens and a side chain), so an important question remains, in what details the ion-binding sites differ in W17’F channels to render them non-conducting.

The dramatically expanded upper pores of the NaK and HCN channels (Fig. 7E,F) are created by an extension and twist of the polypeptide chain similar to that of the inactivated potassium channels (Fig. 7A–C). The first carbonyl oxygen is drawn away from the channel axis through the large reorientation of the Y28’ side chain and its equivalents (D66 in NaK, Y361 in HCN). The second carbonyl, that of G27’ does not face the channel axis but instead faces the adjacent subunit in a clockwise direction in the Kv and NaK channels, but in the counterclockwise direction in HCN (Fig. 7F). In all cases the altered H-bond environment of the residue at 28’ results in a dramatically reoriented sidechain which in turn rotates the top two carbonyls, abolishing the binding sites IS1 and IS2.

### Block of the channel by dendrotoxin

Potassium currents can be inhibited by five distinct families of toxins. The spider toxins Hanatoxin, SGTx1 and VSTx1 interact with the voltage sensors of Kv channels to inhibit voltage-dependent channel activation (Lee & MacKinnon, 2004; Wang et al., 2004). Other toxins bind directly to the extracellular mouth of the channel to block current. The cone-snail toxin Cs1 binds to the extracellular surface of Kv channels and appears to block currents by inducing a conformational change in the protein, collapsing the selectivity filter (Karbat et al., 2019). The scorpion toxin Charybdotoxin is seen to block Kv1.2-paddle chimera channels by inserting a lysine residue directly into the selectivity filter, disrupting the ion-binding site IS1 (Banerjee et al., 2013). The same mechanism holds for block of Kv1.3 by the sea anemone toxin ShK (Selvakumar et al., 2022). Finally, the snake toxin α-Dendrotoxin (DTx) studied here is seen to block Kv1.2 by insertion of a lysine residue into the pore. The DTx mechanism differs from that of CTx and ShK in that the blocking side chain penetrates further into the selectivity filter, occluding site IS2 as well as IS1. We observe that, apart from the modified density in the upper half of the selectivity filter, the remainder of the channel structure is unperturbed, so we conclude that its mechanism of action is a simple plugging of the pore. We failed to see a toxin density in the cryo-EM map when DTx was added to detergent-solubilized Kv1.2-W17’F protein; this is not unexpected as the substantial remodeling of the extracellular vestibule of the channel in the W17’F mutant would eliminate the toxin-binding site.

### The selectivity filter in the absence of K^+^

In the absence of K^+^, large voltage-dependent Na^+^ currents are observed in wildtype Kv2.1 channels (Korn & Ikeda, 1995) and in Shaker channels (Melishchuk et al., 1998). The Kv1.2 structure reported here with Na^+^ replacing all K^+^ in the solution shows a selectivity filter conformation that at first glance is unchanged from the K^+^ structure (Figure 5B). Ion densities are seen in the ion-binding sites, but these appear to be Na^+^ ion densities, and the carbonyl-carbonyl distances in the binding sites are narrowed as would be expected for the coordination of the smaller Na^+^ ions.

The Shaker-W17’F channel in the absence of K^+^ stably carries large Na^+^ and Li^+^ currents (Starkus et al., 1997). These currents are blocked by low concentrations of K^+^, and this would be expected as potassium binding to the sites IS3 and IS4 in this mutant is very tight (Tan et al., 2022). We therefore sought to obtain the structure of the corresponding Kv1.2-W17’F channel in Na^+^ solution (Figure 6). In 2D classes the complex appeared to be highly unstable, with large variations in the “wobble” angle between the TMD and the intracellular domains. The intracellular domains could be reconstructed to 3.0 Å resolution, but attempts to recover the structure of the transmembrane region by focused refinement yielded only low-resolution structural information. We conclude that this channel, at least as solubilized in detergent, has a highly unstable conformation when K^+^ ions are absent.

## Methods

### Kv1.2-Kvβ expression and purification

The rat Kv1.2 alpha-subunit constructs were derived from that of Long et al. (2005), having the full-length (GenBank: X16003) sequence containing the mutation N207Q to eliminate a glycosylation site. Our “native” construct contained the additional mutations L15H and G198S in unstructured regions; the inactivating construct contained the further mutation W366F. To each of these was added at the N-terminus tandem Strep tags (sequence WSHPQFEK) separated by a Gly-Ser linker. The beta-subunit construct was derived from the rat beta2 core, residues 36–357 (Gulbis et al., 1999). To eliminate very strong interactions between the many Lys residues on the “bottom” of the beta-subunits and negatively-charged carbon film or graphene substrates, we mutated to glutamine the five lysine residues at positions 94, 104–106 and 258. The fold of the beta subunits containing these mutations is indistinguishable from the wildtype structure (Long et al., 2007).

To express both subunits in a single construct, Kv1.2 and Kvβ genes were separately inserted into pPicZ-B (Thermo-Fisher) vectors between the XhoI and AgeI sites in the multi-restriction-sites region. Subsequently the pPicZ-B vector carrying Kvβ was opened at BglII and BamHI restriction sites, and the insert including the AOX1 promoter was inserted into the other pPicZ-B vector, upstream of the AOX1-driven Kv1.2 cDNA, at the BglII site.

The *P. pastoris* strain SMD1168 (Invitrogen) was electroporated with the pPicZ-B plasmids. YPDS (yeast extract, peptone, dextrose, and sorbitol) plates containing Zeocin (800 ug/ml) were used to select transformants. Stocks were stored in 15% glycerol at −80°C.

The expression and purification procedures were based on those of Long et al. (Long et al., 2005). Briefly, we inoculated 1 liter of BMGY medium (this and the other yeast media were obtained from Invitrogen) with 10 ml of an overnight culture grown in YPD medium with Zeocin (100 ug/ml). After centrifugation (5500× *g*, 10 minutes), the cells were transferred to BMMY medium including 0.5% (v/v) methanol and grown for 24 h. After adding an additional 0.5% methanol to increase protein expression, cells were grown for 2–3 days. Cells were centrifuged (5500× *g*, 20 minutes), the pellet was frozen and stored at −80°C.

Thawed cells were lysed at 4° in a French Press. One gram of cell lysate was resuspended in 5mL of Lysis buffer consists of 100 mM Tris-HCl (pH 8.0), 150 mM KCl, 300 mM sucrose, 5 mM EDTA, 0.05 mg deoxyribonuclease I, 1 mM MgCl2, 1 mM phenylmethylsulfonyl fluoride, 1 mg/ml each of leupeptin, pepstatin and aprotin, and 0.1 mg/mL of soy trypsin inhibitor. Lysis buffer plus 30 mM dodecylmaltoside (DDM) was used to solubilize the membranes for 3 hours at room temperature. Centrifugation (13000× *g*, 20 minutes) separated the unsolubilized material. The supernatant was added to Strep-Tactin Sepharose beads (IBA) preequilibrated with Membrane Resuspension Buffer (100 mM Tris-HCl pH 8.0, 150 mM KCl, 3mM TCEP, 5 mM EDTA, 10 mM beta-mercaptoethanol, and 5 mM DDM. The bead slurry (approximately 1 ml) was incubated 1h at 4°C with gentle rotation. A column was used to collect the beads after incubation, followed by washing with 14 volumes of Membrane Resuspension Buffer with added lipids (0.1 mg/ml of the mixture 3:1:1 of POPC:POPE:POPG). The addition of 10 mM desthiobiotin was used to elute the bound protein. The eluted protein was concentrated with a Millipore Amicon Ultra 100 K filter and further purified by size-exclusion chromatography (SEC) on a Superose S6 column pre-equilibrated with 20 mM tris-HCl (pH 7.5), 150 mM KCl, 2 mM TCEP, 10 mM DTT, 1 mM EDTA, 1 mM PMSF, 1 mM dodecylmaltoside (Anatrace, anagrade) and 0.1 mg/ml lipid mixture (3:1:1 of POPC:POPE:POPG). We pooled fractions containing both alpha and beta subunits, and when necessary concentrated the protein to 1~2 mg/ml (Amicon Ultra 100 KDa, Millipore). For experiments under K^+^-free conditions, in the SEC buffer NaCl replaced KCl.

### Oocyte expression and voltage-clamp recordings

The native or W366F Kv1.2 alpha subunit construct was cloned into a pcDNA3.1 vector. RNA was prepared from the XbaI-linearized plasmid using T7 RNA polymerase. Xenopus oocytes were defolliculated by collagenase treatment, injected with cRNA and stored in ND96 solution (96 mM NaCl, 2 mM KCl, 1.8 mM CaCl2, 1 mM MgCl_2_, 5 mM HEPES, pH 7.4 with NaOH) at 18°C. Recordings were done at room temperature, 5–6 days post-injection in ND96 solution or KD96, the same solution but made with 96 mM KCl, 2 mM NaCl and KOH. Two-electrode voltage clamp recordings employed an OC-725C amplifier (Warner Instruments).

### Cryo-EM specimen preparation, data acquisition and processing

Quantifoil holey carbon grids (R1.2/1.3, 300 mesh, Au) were glow-discharged under vacuum at 15 mA for 1 min with carbon side facing upwards in the chamber. The chamber of the freezing apparatus (Vitrobot Mark IV, Thermo Fisher Scientific) was preequilibated at 16 °C and 100% humidity. A 3 μL droplet of protein sample was applied to the carbon side of each grid and blotted for 3 or 5 s with zero blotting force after 15 s wait time before plunge-freezing in liquid ethane. For the DTX bound sample, α-DTX (Alomone Labs) was mixed with Kv1.2 WT protein at a 100 nM final concentration 30 min prior to cryo-EM grid preparation.

For the Kv1.2 WT-Na^+^, W366F-Na^+^, and DTX-bound samples, graphene-covered grids were used to allow lower protein concentrations (0.05 to 0.1 mg/ml) to be used. These were prepared from commercial graphene (Trivial Transfer Graphene, single layer; ACS Material) using the recommended transfer protocol onto the quantifoil grids, which were then plasma-cleaned (Gatan 950 Advanced Plasma System) under Ar/O_2_ at 10 W for 10 s with the graphene coated surface facing upwards in the chamber. Vitrification was conducted similarly with 30 s wait time.

Cryo-EM samples were imaged on a Titan Krios cryo-EM (Thermo Fisher Scientific) operated at 300 keV, equipped with a Bio Quantum energy filter (20 eV slit width) and K3 camera (Gatan). Micrographs were collected using super-resolution mode, physical pixel size 1.068 Å, with SerialEM software (Schorb et al., 2019) using image shift patterns and one shot per hole. A total of 6335, 8310, 4507, 2572, 4680 micrographs were collected for WT, W366F, DTX-bound, Na^+^ bound WT, and Na^+^ bound W366F, datasets respectively.

Processing for each dataset is summarized in the Figure Supplements to Figs. 1–4. Beam-induced motion was corrected by MotionCor2 (Zheng et al., 2017) and CTF values were estimated by Gctf (Zhang, 2016). Particles were picked either by reference-free Laplacian-of-Gaussian autopicking; an adversarial-template-based program Demo Picker written by F.J.S.; or RELION reference-based auto-picking. All further processing used RELION 3.1. Picked particles were extracted and subjected to rounds of 2D and 3D classification before refinement. Transmembrane-domain masks were used for focused refinement to optimize the resolution of the transmembrane region. Beam-tilt and per-particle CTF refinement followed by per-particle motion correction were applied to further improve the resolution.

### Protein model building, refinement and structural analysis

An Alpha-Fold predicted rat Kv1.2 model (alphafold.ebi.ac.uk/entry/Q09081) was used to build the Kv1.2 native atomic model. The W366F, DTX-bound, and Na^+^-bound models were subsequently built from this Kv1.2 model. Model fitting was performed with the CCPEM program suite. Initial docking was performed with Molrep; obvious outliers of were manually fixed in Coot 0.9 (Emsley et al., 2010) and real space refinement used Refmac 5 (Murshudov et al., 2011) and Phenix (Liebschner et al., 2019). Density map rendering, analysis and figure preparation were done with UCSF Chimera and ChimeraX (Goddard et al., 2018; Pettersen et al., 2004).

## Supplementary Material

Supplement 1
