# Supplementary material for: Cryo-EM structures of Kv1.2 potassium channels, conducting and non-conducting
Source: bioRxiv. 2024 Mar 19:2023.06.02.543446. Originally published 2023 Jun 3. Preprint. [Version 2] doi: 10.1101/2023.06.02.543446 (PMC10312591; doi:10.1101/2023.06.02.543446)
Supplement: Supplementary file 1 [file NIHPP2023.06.02.543446v2-supplement-1.pdf]

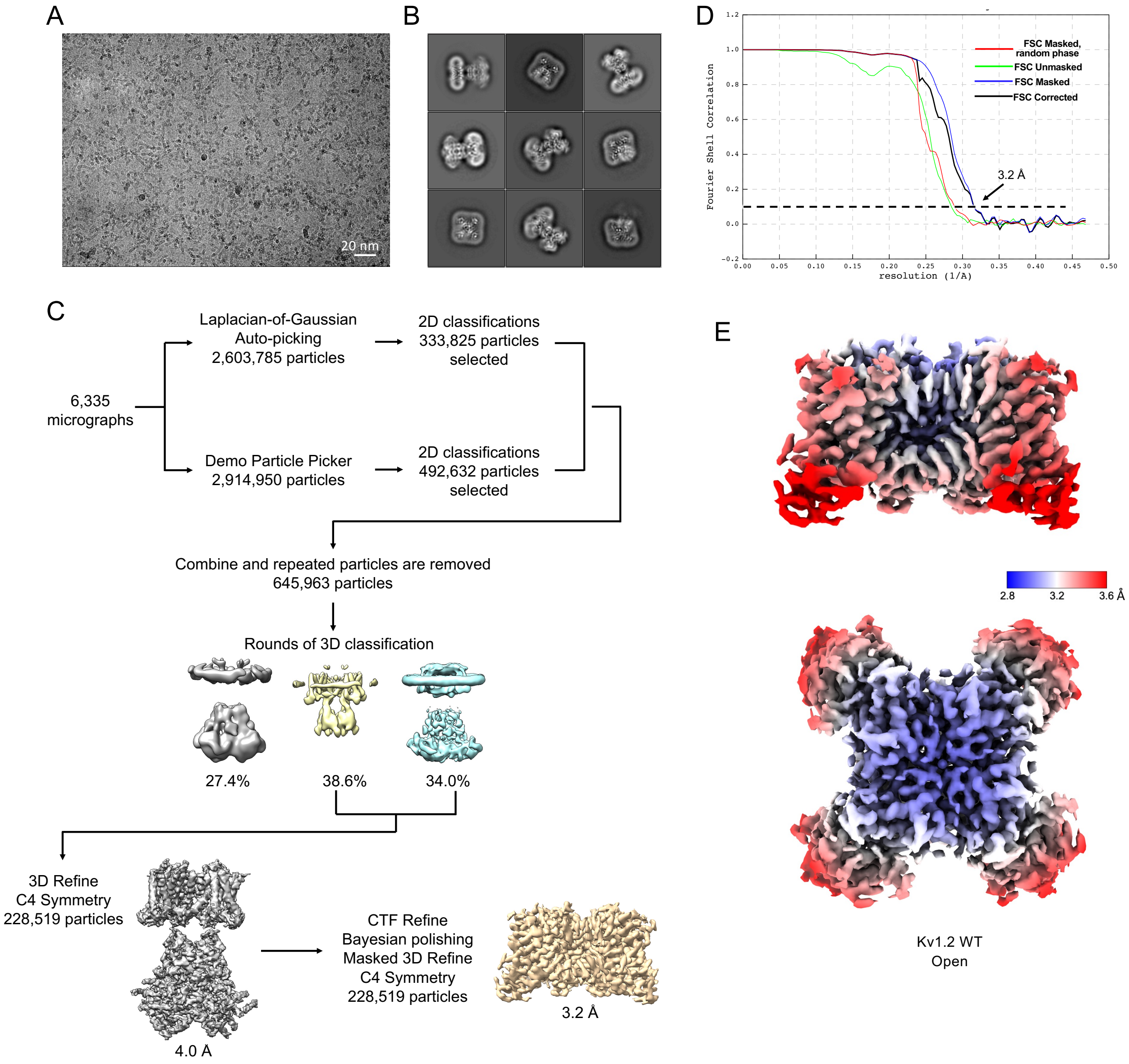

Figure 1 - figure supplement 1. Image processing and reconstruction of Kv1.2$_S$. (A) Representative micrograph. (B) Representative 2D classes. (C) Cryo-EM data processing workflow. (D) Gold standard FSC resolution estimation. (F) Local resolution estimation.

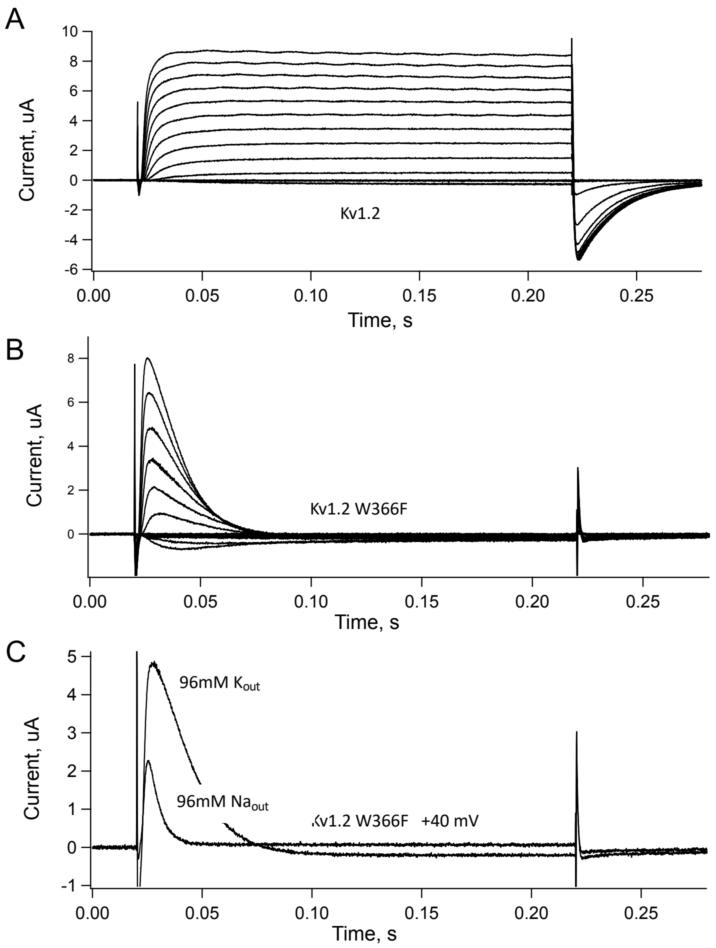

Figure 2 - figure supplement 1, currents from native and W366F Kv1.2 channels. *Xenopus* oocytes were injected with mRNA for the alpha subunit constructs used in this study. **A**, native Kv1.2 currents elicited from pulses to -60 to + 60mV in 10 mV steps, from a holding potential of -80mV. **B**, Same voltage protocol applied to channels with W366F alpha subunits, recorded with 96 mM K^+ bath solution. **C**, Comparison of currents elicited from an oocyte at +40 mV with 96mM K^+ or Na^+ bath solutions. The potassium-free Na^+ solution yielded faster inactivation, as expected for C-type inactivation. The apparently sustained current in 95 mM Na^+ solution is an artifact of P/4 leak subtraction at -120mV holding potential.

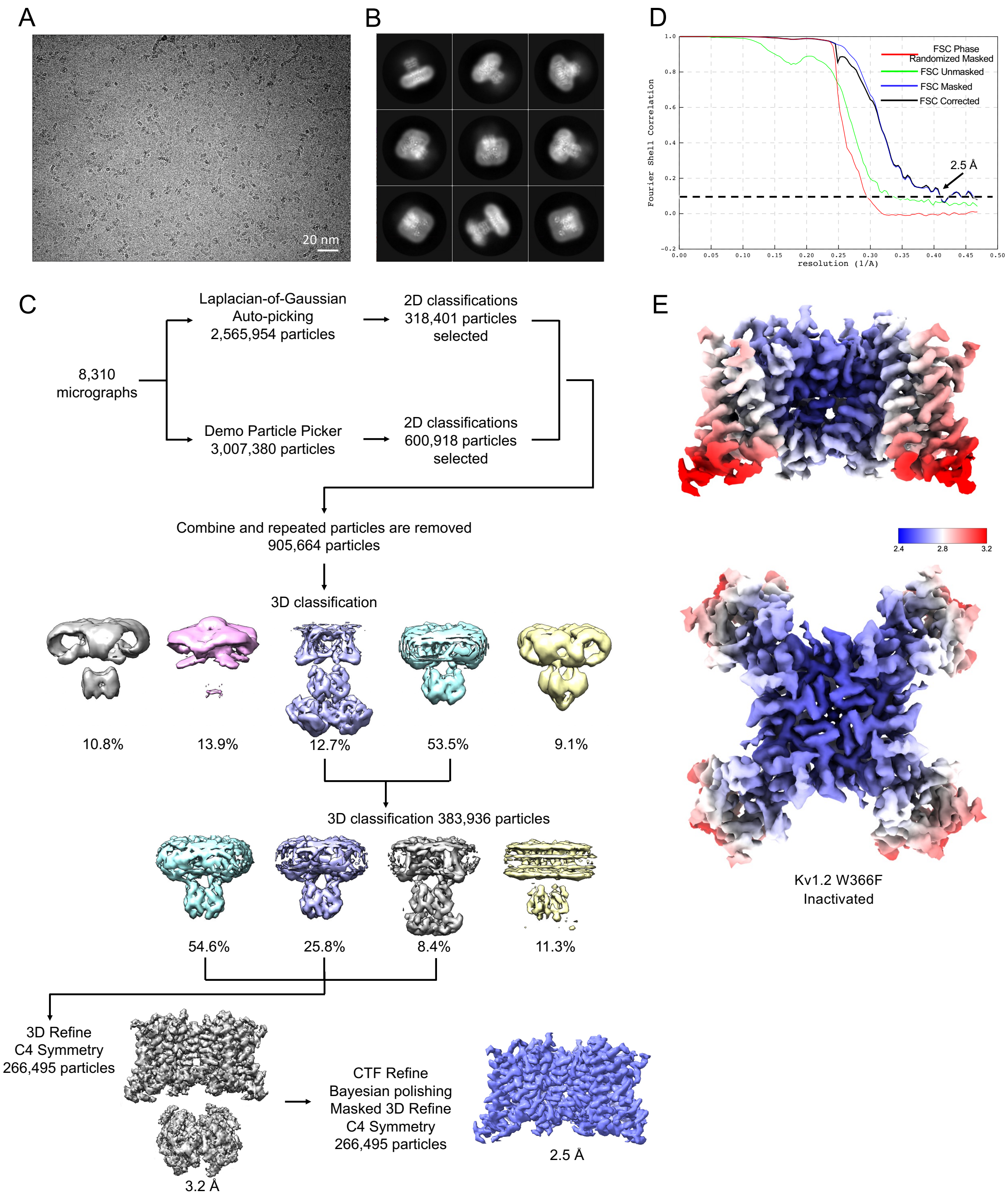

Figure 2 - figure supplement 2, Processing of Kv1.2 W366F images. (A) Representative micrograph. (B) Representative 2D classes. (C) Cryo-EM data processing workflow. (D) Gold standard FSC resolution estimation. (F) Local resolution estimation.

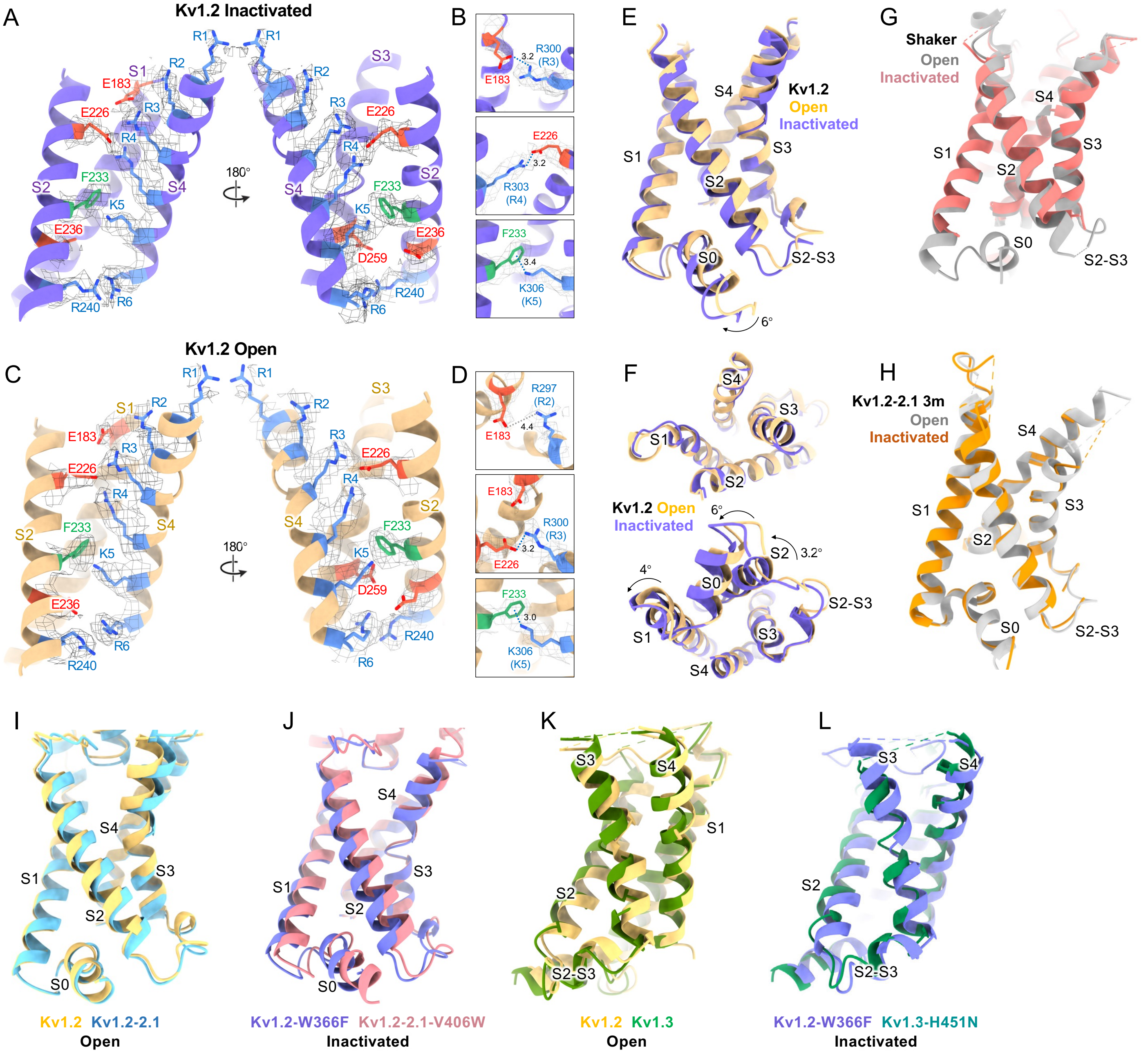

Figure 2 - figure supplement 3. Voltage-sensing-domain conformational differences between open and C-type inactivated states. Side view of VSD structures and maps of Kv1.2$_s$ in (A) inactivated state. (B) Kv1.2$_s$ VSD R3/E183 (upper), R4/E226 (middle), and K5/F233 (lower) interactions in the inactivated state. VSD structure in open state. (D) VSD relative position of R2/E138 (upper) and R3/E226 (middle), K5/F233 (lower) interactions in the open state. Positively charged and negatively charged residues are shown in blue and red, while aromatic residues are shown in green. Side view (E), top view (F, upper) and bottom view (F, lower) VSD conformational difference between open (yellow) and inactivated (purple) states. Superposition of (G) Shaker open (PDB: 7SIP), Shaker W434F inactivated (PDB: 7SJ1) and (F) Kv1.2-2.1 open (PDB: 7SIZ), Kv1.2-2.1 3m inactivated (PDB: 7SIT) VSD structures. (I-L) Superposition of VSD structures. (I) Kv1.2$_s$ (yellow) and Kv1.2-2.1 (light blue, PDB: 2R9R); (J) Kv1.2$_s$ W366F (purple) and Kv1.2-2.1 V406W (carnation, PDB: 5WIE); (K) Kv1.2 (yellow) and Kv1.3 (green, PDB: 7EJ1); (L) Kv1.2 W366F (purple) and Kv1.3 H451N (clover, PDB: 7EJ2).

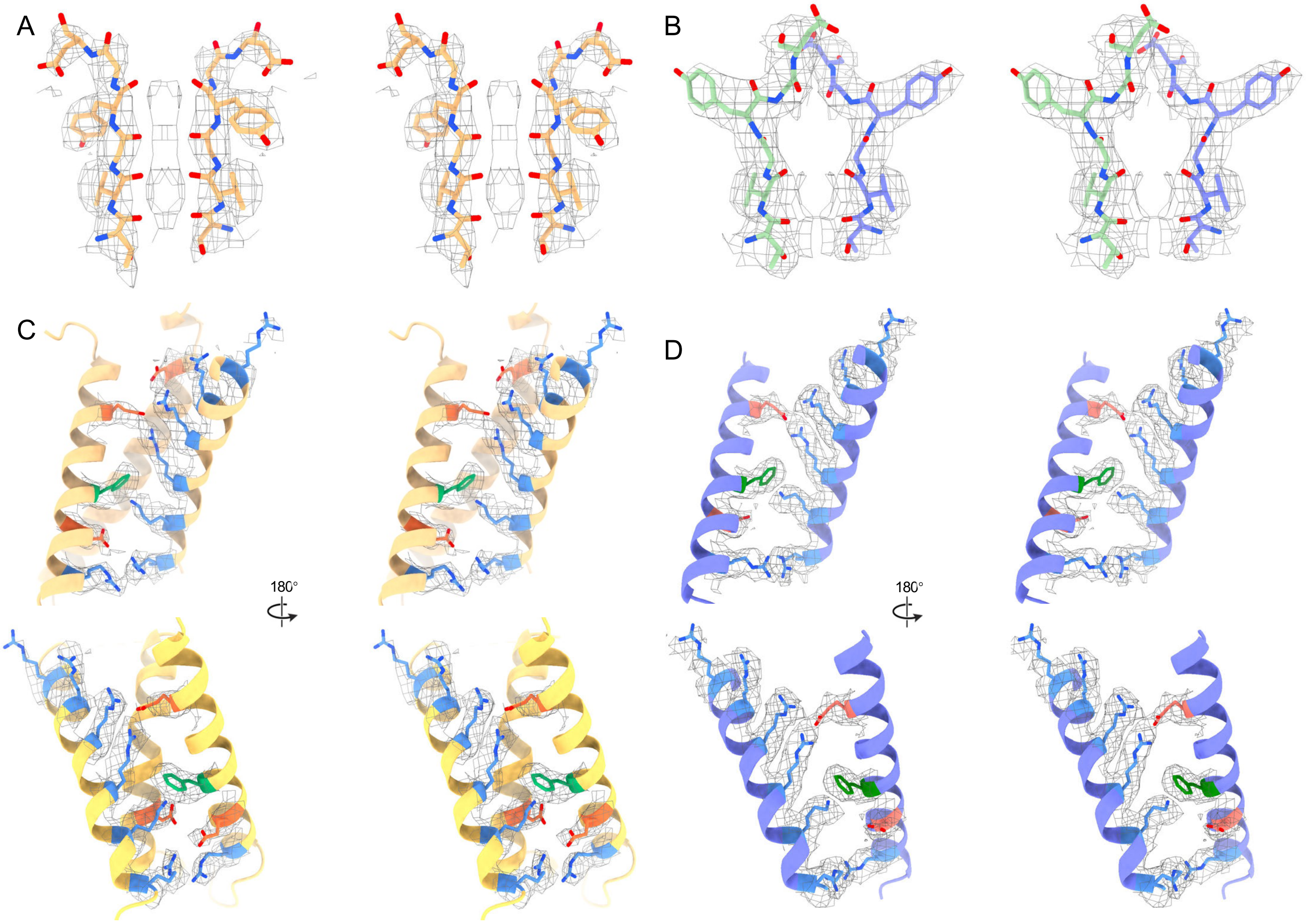

Figure 2 - figure supplement 4 Stereo views of selectivity filter and voltage-sensing-domain.
Stereo view of (A) Kv1.2s SF, (B) Kv1.2s-W366F SF, (C) Kv1.2s VSD, (D) Kv1.2s-W366F VSD.

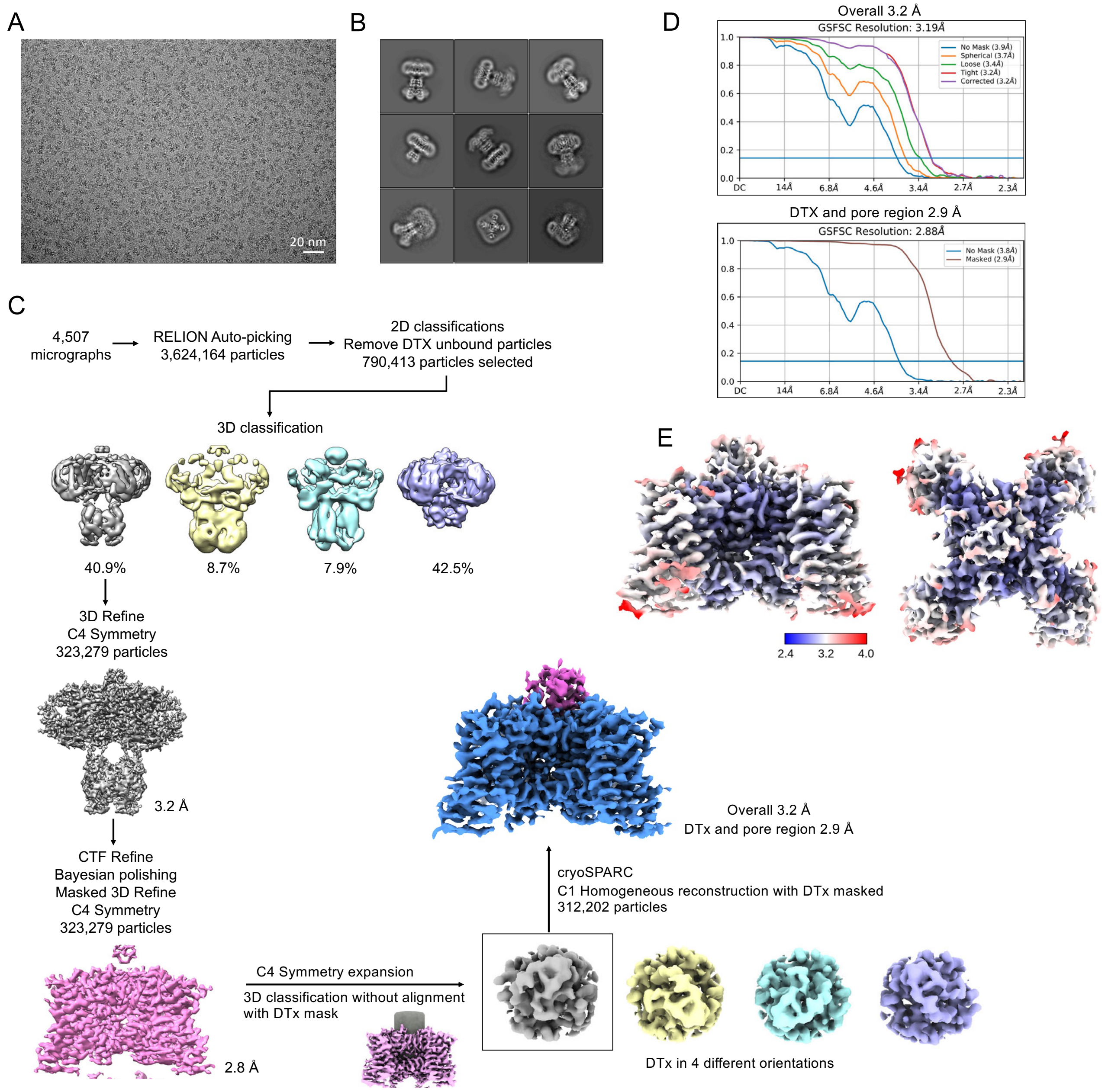

Figure 3 - figure supplement 1. Cryo-EM imaging and reconstruction of Kv1.2$_S$-DTX. (A) Representative micrograph. (B) Representative 2D classes, showing the DTx “cap” on the particles. (C) Cryo-EM data processing workflow. See Methods for details of the symmetry expansion and C1 reconstruction. (D) Gold standard FSC resolution estimation for the overall map (top) and the DTX-plus-selectivity filter masked region. (E) Local resolution estimation.

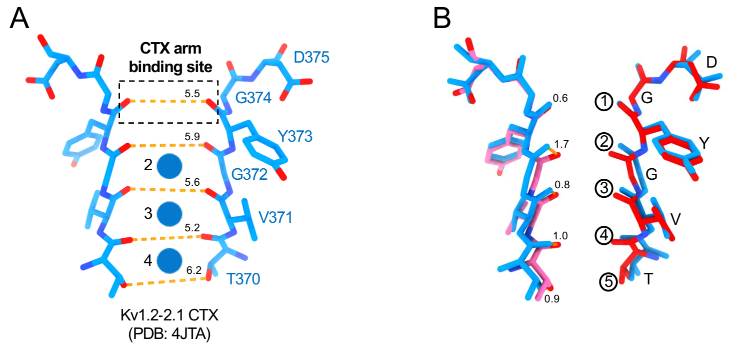

Figure 3 - figure supplement 2 Comparison of the Kv1.2-2.1 CTx bound selectivity filter with the Kv1.2$_S$ DTx-bound structure. (A) Side view of the selectivity filter of Kv1.2-2.1 CTx bound conformation (Banerjee et al. 2013). Orange dashed lines show the distances between carbonyl oxygens, for comparison with Fig. 3G. Potassium ions are shown as blue balls. (B) Superposition of Kv1.2 DTx (red) and Kv1.2-2.1 CTx (blue) selectivity filter structures. Apparent carbonyl displacements are given in Å.

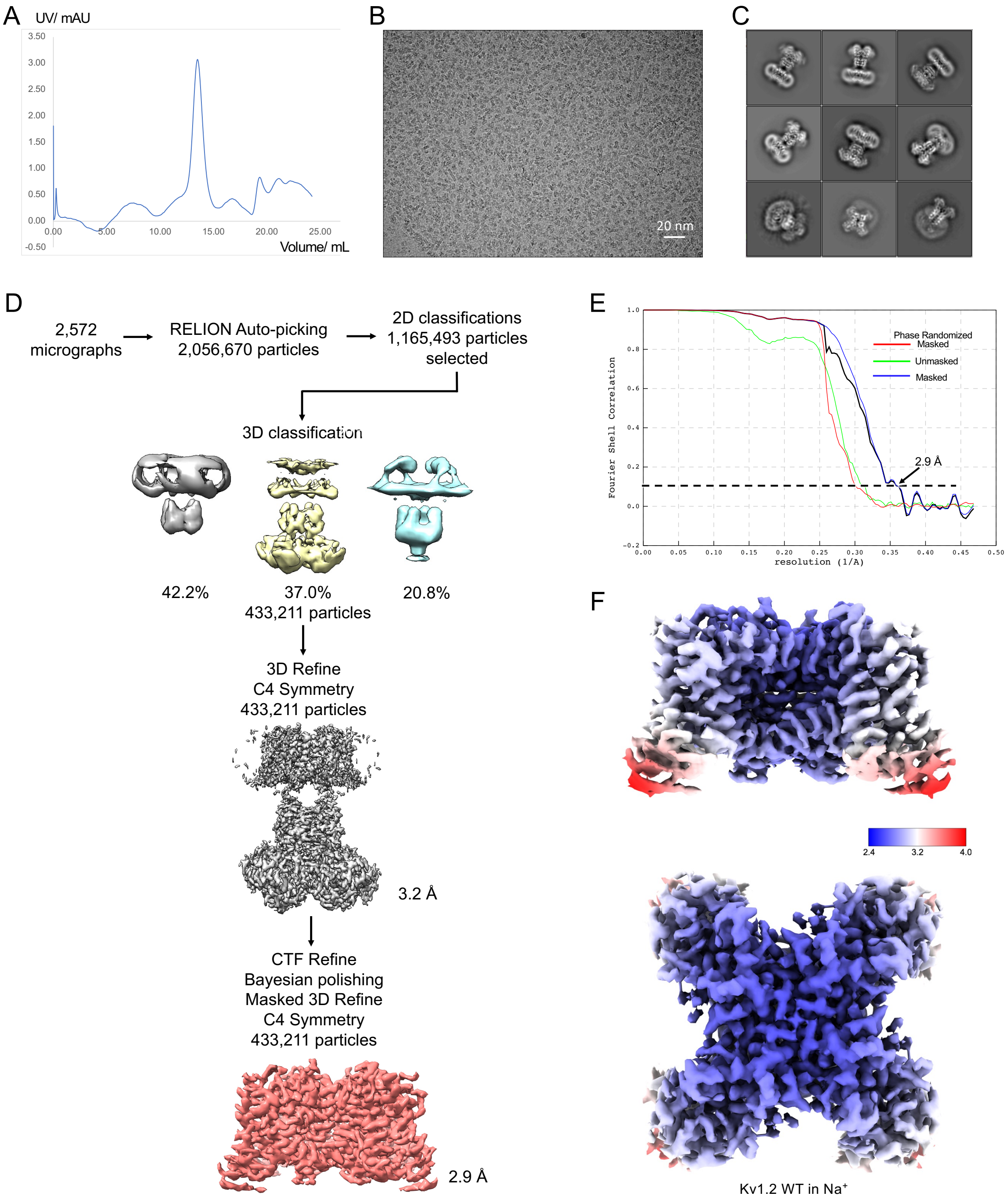

Figure 4 - figure supplement 1 Cryo-EM of Kv1.2$_S$ in Na$^+$. (A) Size-exclusion chromatogram. Detector drift was large compared to a small protein signal. (B) Representative micrograph showing monodisperse particles on the graphene substrate. (C) Representative 2D classes. (D) Cryo-EM data processing workflow. (E) Gold standard FSC resolution estimation. (F) Local resolution estimation.

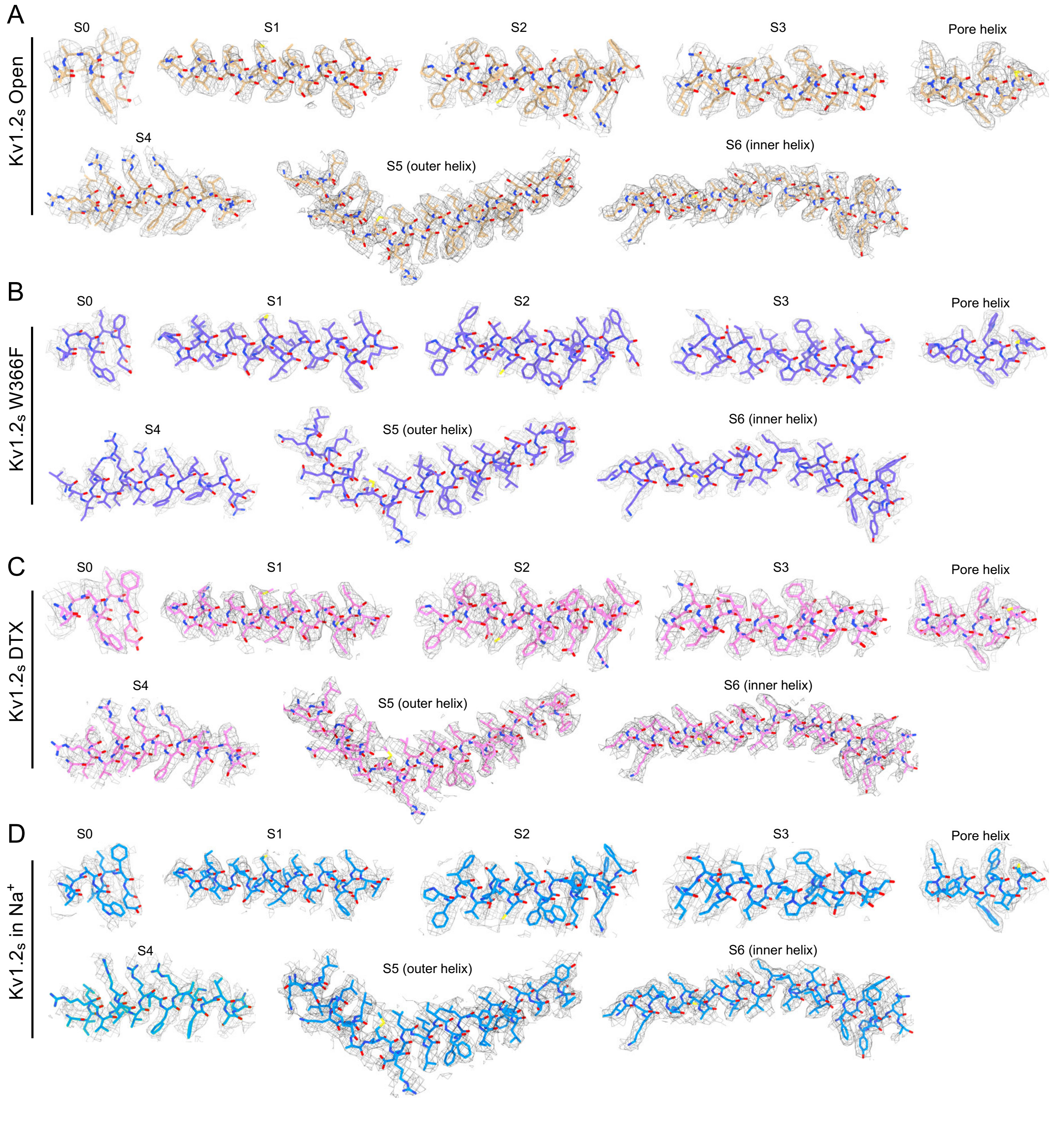

Figure 4 - figure supplement 2. Comparisons of cryo-EM density map and model for alpha helices in each Kv1.2 structure reported here.

	Kv1.2-open	Kv1.2-W366F	Kv1.2-DTx	Kv1.2-Na+	Kv1.2-W366F-Na+
**Data collection and processing**					
Microscope			FEI Titan Krios		
Detector			K3		
Voltage (kV)			300		
Electron exposure (e-/Å^2)			50		
Magnification			81000		
Defocus range (um)			-1.0 to -2.0		
Pixel Size (Å/pixel)			1.068		
Symmetry imposed	C4	C4	C1	C4	C4
Micrographs (no.)	6335	8310	4507	2573	9628
Map resolution (Å)	3.2	2.5	3.2	2.8	7.8
Final particle images (no.)	228519	266495	312202	433211	239100
FSC threshold			0.143		
**Refinement**					
PDB codes	6VC6	6VCH	6VC3	6VC4	
EMDB codes	43134	43136	43131	43133	
Map sharpening B factor (Å^2)	-185.2	-50	-116.8	-157.8	
Model Composition					
Chains	4	5	6	4	
Atoms	7740	7614	8219	7740	
Residues	1028	1012	1087	1028	
Water	0	0	0	0	
Ligands	0	K: 2	K: 2	0	
Bonds (RMSD)					
Length (Å)	0.003 (0)	0.004 (0)	0.002 (0)	0.003 (0)	
Angles (°)	0.562 (0)	0.577 (0)	0.484 (0)	0.621 (0)	
Validation					
Molprobity score	1.52	1.37	1.43	1.55	
Clashscore	7.58	5.83	7.03	8.1	
Poor rotamers (%)	0.26	0.65	0.24	0.56	
Ramachandran plot					
Favoured (%)	97.51	97.77	97.83	97.91	
Allowed (%)	2.49	2.23	2.17	2.09	
Outliers (%)	0.00	0.00	0.00	0.00	

Figure 4 - figure supplement 3 Cryo-EM data collection, refinement and validation statistics

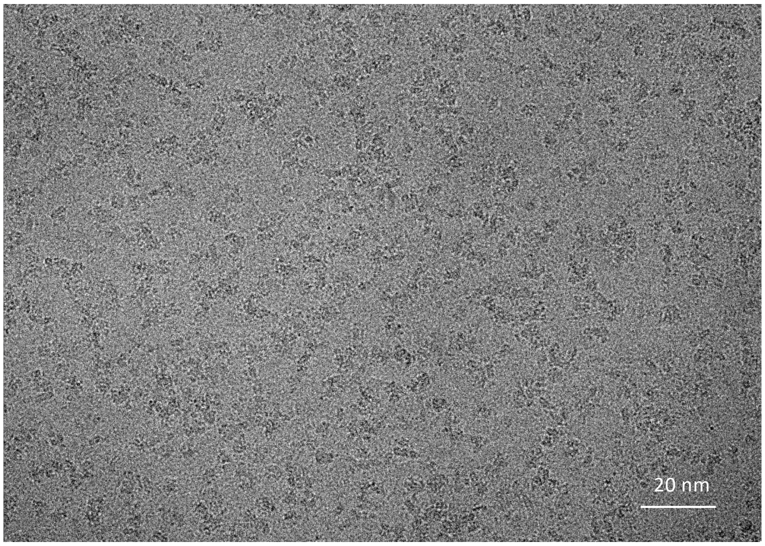

Figure 5 - figure supplement 1 Representative micrograph of Kv1.2 W366F in Na^+, demonstrating the absence of protein aggregates on the graphene substrate.

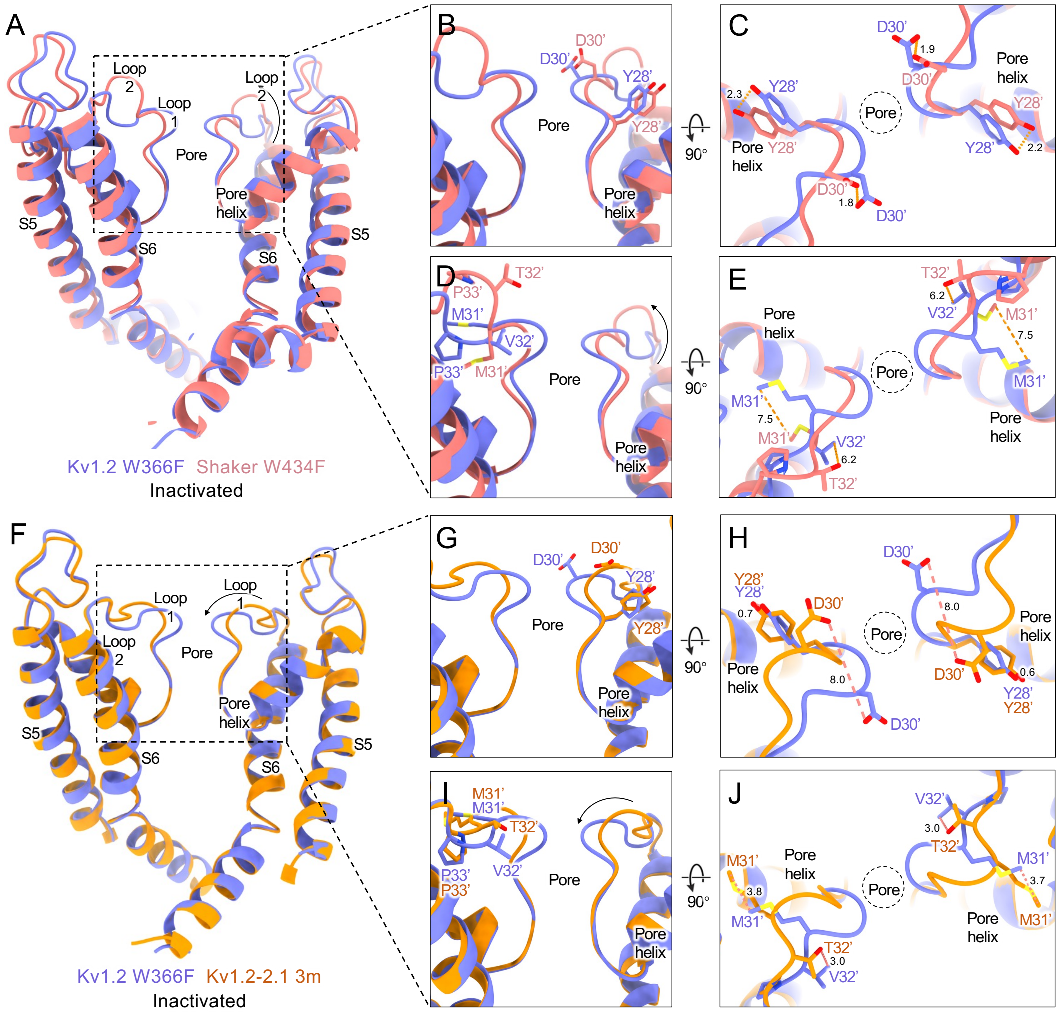

K	Asp	Val/Thr	Loop1	Loop2	H-bonding
Kv1.2 W366F	D28'-flipped	V32'	unflipped	flipped	Inter/intra-subunit
Shaker W434F	D28'-flipped	T32'	unflipped	flipped	Intra-subunit
Kv1.2-2.1 3m	D28'-not flipped	T32'	flipped	unflipped	Inter-subunit

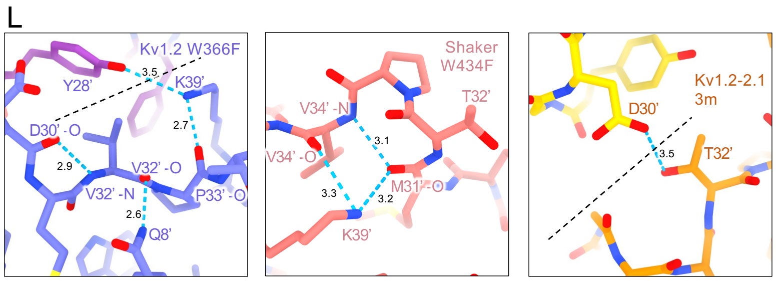

Figure 6–Figure Supplement 1. Structural comparison of inactivated Kv channels. (A-J) Structural superposition of Kv1.2$_s$-W366F pore domain with other inactivated channels: side view with Shaker W434F (A) or Kv1.2-2.1-3m (F). Loop 1 conformational differences with Shaker W434F side view (B), top view (C); or with Kv1.2-2.1-3m side view (G), top view (H). Loop 2 conformational changes with Shaker W434F side view (D), top view (E); Kv1.2-2.1 3m side view (I), top view (J). (K) Table lists of the differences among the inactivated Kv channels. (L) H-bonds among the inactivated Kv channels. Adjacent subunits are shown as different colors, and a dashed black line denotes the subunit boundary. H-bonding patterns affect the stability of the inactivated state and an obvious difference is at the location 32', where Shaker has Thr and Kv1.2 has Val. The mutation V32'T in the Kv1.2-2.1 background provides a new hydrogen bond that stabilizes the important residue D30' in the inactivated conformation; this can be seen in the Kv1.2-2.1-3m structure (L, panel 3). Our inactivated Kv1.2s structure (containing V32') nevertheless shows an H-bond network that includes Y28' and D30' along with main-chain atoms, possibly yielding a similar stabilization of the inactivated state (L, panel 1). The inactivated Shaker structure lacks H-bond partners for either Y28' or D30', which instead are exposed to solvent; however another H-bond network stabilizes the P-loop-S6 linker (L panel 2).

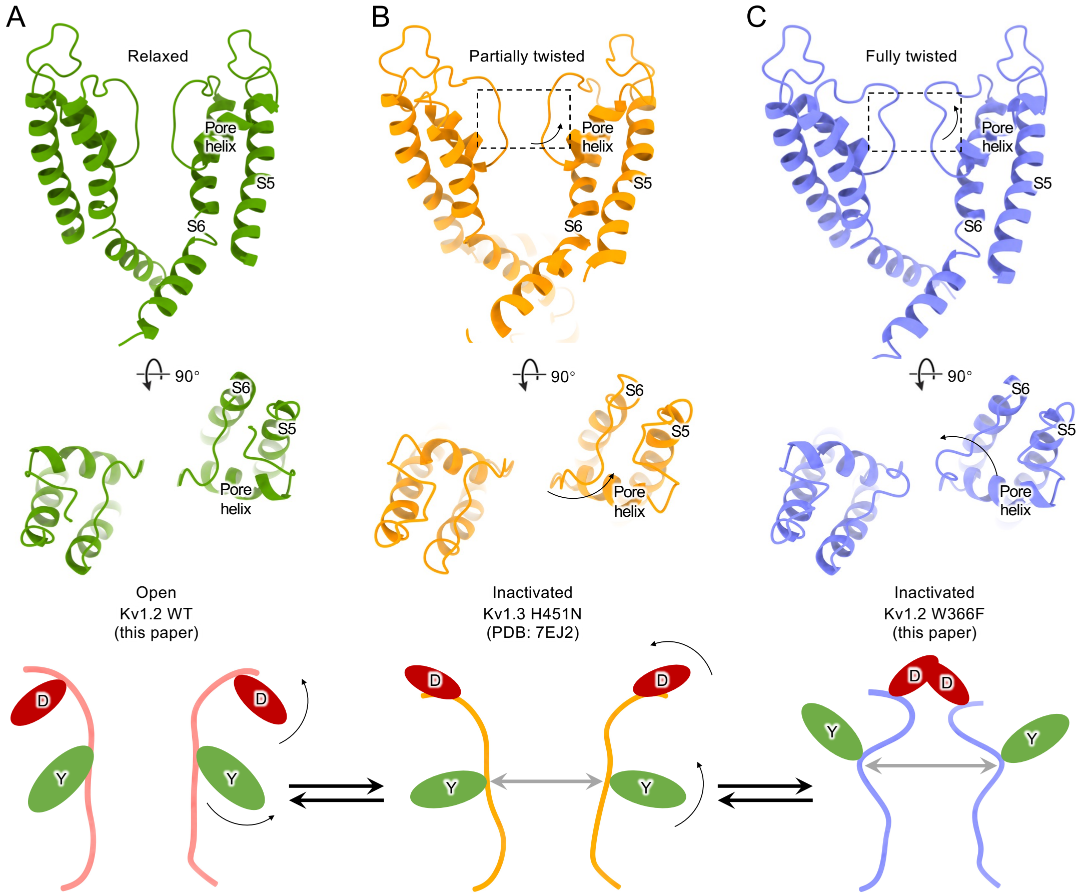

Figure 6 - figure supplement 2. Summary of conformational changes in Kv channel inactivation. Upper panels: (A) Kv1.2 WT pore domain (PD) in green (B) Kv1.3 H451N PD in orange and (C) Kv1.2 W366F PD in orchid represent the relaxed, partially twisted and fully twisted P-loop respectively. Lower panels: cartoon illustration of (A) relaxed, (B) partially twisted and (C) fully twisted selectivity filter P-loop of Kv channels. D30' and Y28' residue side chains are shown as red and green ovals.
