# Supplementary material for: Cryo-EM structures of Kv1.2 potassium channels, conducting and non-conducting
Source: bioRxiv. 2023 Jun 3:2023.06.02.543446. Preprint. [Version 1] doi: 10.1101/2023.06.02.543446 (PMC10312591; doi:10.1101/2023.06.02.543446)
Supplement: Supplementary file 1 [file NIHPP2023.06.02.543446v1-supplement-1.pdf]

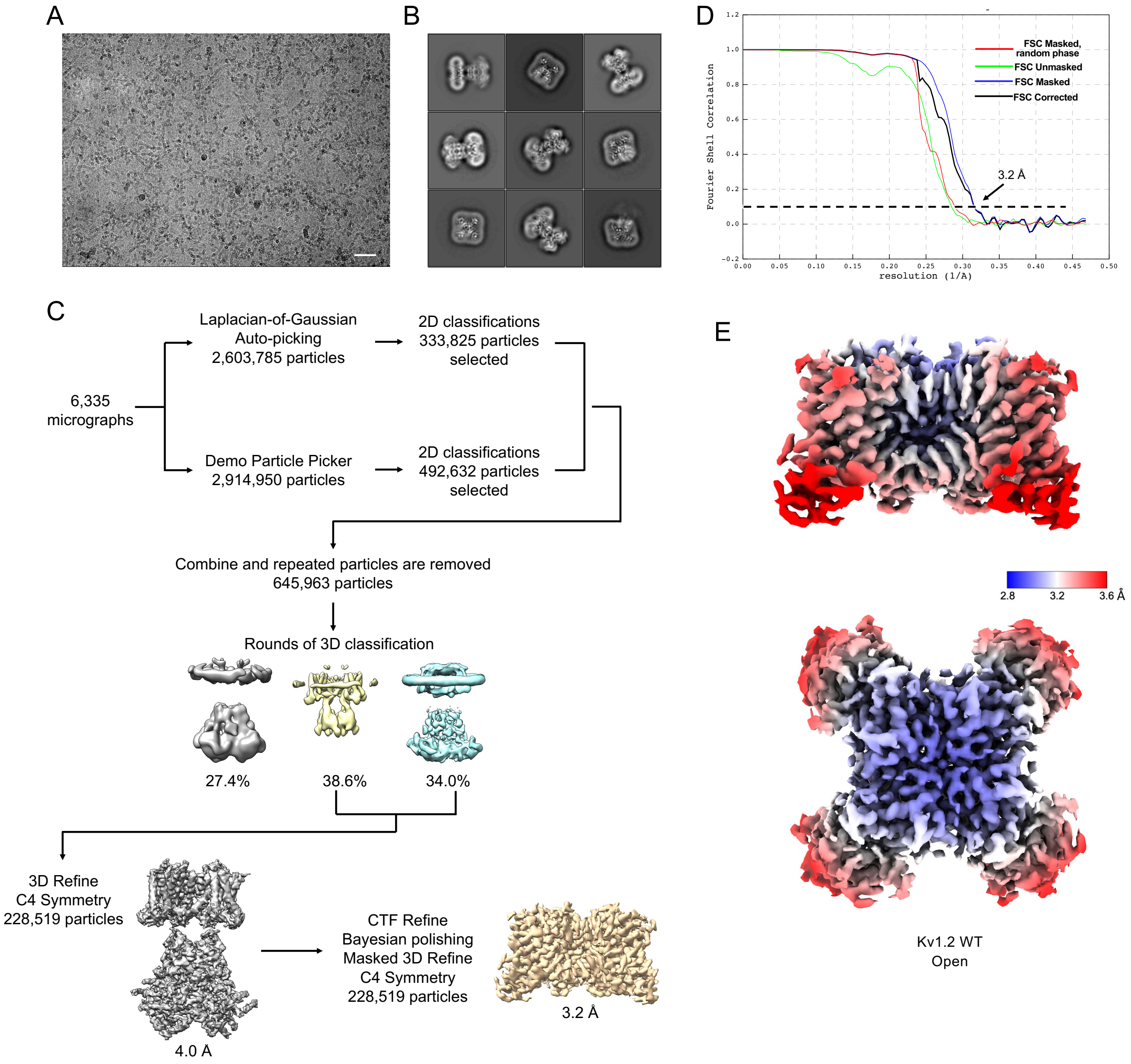


**Figure 1 - figure supplement 1, image processing and reconstruction of native Kv1.2.** (A) Representative micrograph. (scale bar 20 nm) (B) Representative 2D classes. (C) Cryo-EM data processing workflow. (D) Gold standard FSC resolution estimation. (F) Local resolution estimation.

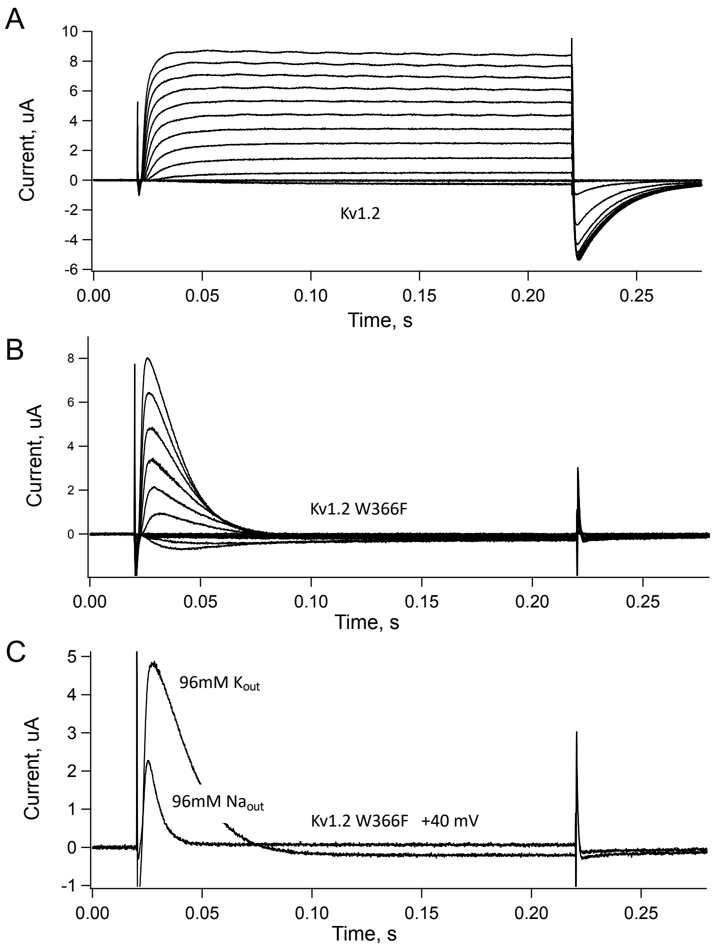


**Figure 2 - figure supplement 1, currents from native and W366F Kv1.2 channels.** *Xenopus* oocytes were injected with mRNA for the alpha subunit constructs used in this study. **A**, native Kv1.2 currents elicited from pulses to -60 to + 60mV in 10 mV steps, from a holding potential of -80mV. **B**, Same voltage protocol applied to channels with W366F alpha subunits, recorded with 96 mM $K^+$ bath solution. **C**, Comparison of currents elicited from an oocyte at +40 mV with 96mM $K^+$ or $Na^+$ bath solutions. The **low K** potassium-free $Na^+$ solution yielded faster inactivation, as expected for C-type inactivation. The apparently sustained current in 95 mM $Na^+$ solution is an artifact of P/4 leak subtraction at -120mV holding potential.

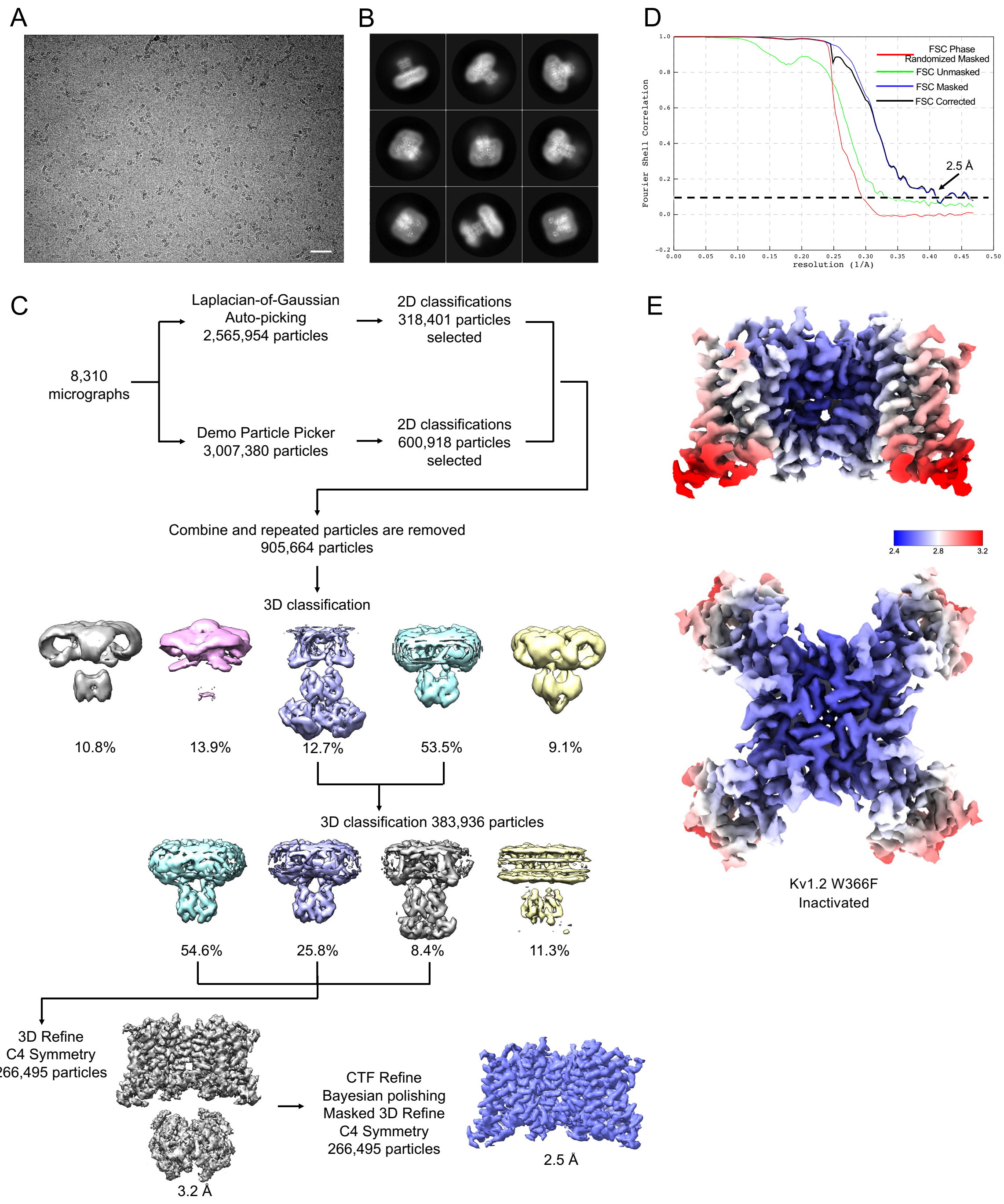


**Figure 2 - figure supplement 2, Processing of Kv1.2 W366F images.** (A) Representative micrograph. (scale bar 20 nm) (B) Representative 2D classes. (C) Cryo-EM data processing workflow. (D) Gold standard FSC resolution estimation. (F) Local resolution estimation.

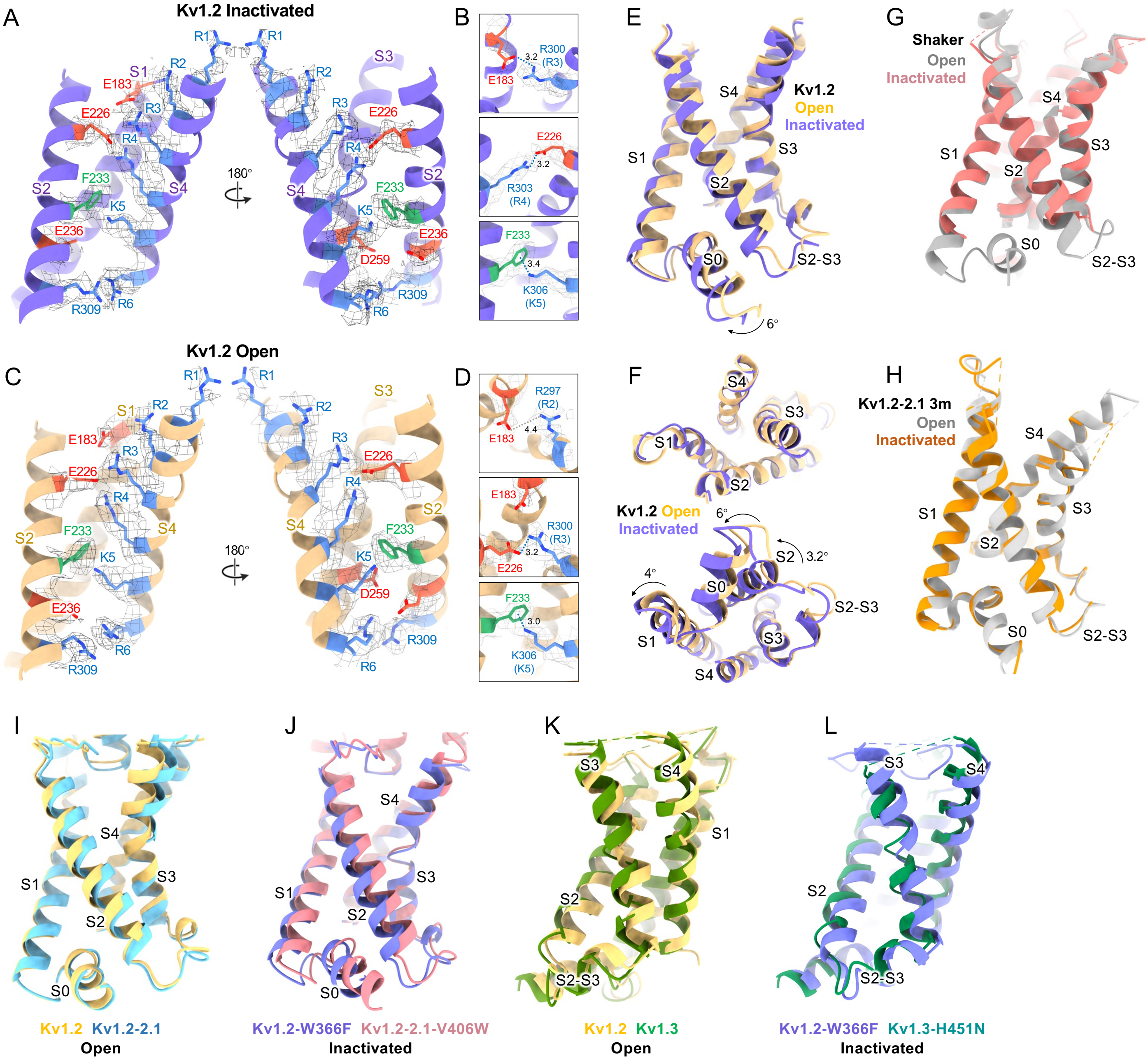


**Figure 2 - figure supplement 3 Voltage sensing domain conformational differences between open and C-type inactivated states.** Side view of VSD structures and maps of Kv1.2 in (A) inactivated state (purple). (B) Kv1.2 VSD R3/E183 (upper), R4/E226 (middle), and K5/F233 (lower) interactions in the inactivated state. VSD structure in open state (yellow). (D) VSD relative position of R2/E138 (upper) and R3/E226 (middle), K5/F233 (lower) interactions in the open state. Positively charged and negatively charged residues are shown in blue and red, while aromatic residues are shown in green. Side view (E), top view (F, upper) and bottom view (F, lower) VSD conformational difference between open (yellow) and inactivated (purple) states. Superposition of (G) Shaker open (PDB: 7SIP), Shaker W434F inactivated (PDB: 7SJ1) and (F) Kv1.2-2.1 open (PDB: 7SIZ), Kv1.2-2.1 3m inactivated (PDB: 7SIT) VSD structures. (I-L) Superposition of VSD structures. (I) Kv1.2 (yellow) and Kv1.2-2.1 (light blue, PDB: 2R9R); (J) Kv1.2 W366F (purple) and Kv1.2-2.1 V406W (carnation, PDB: 5WIE); (K) Kv1.2 (yellow) and Kv1.3 (green, PDB: 7EJ1); (L) Kv1.2 W366F (purple) and Kv1.3 H451N (clover, PDB: 7EJ2).

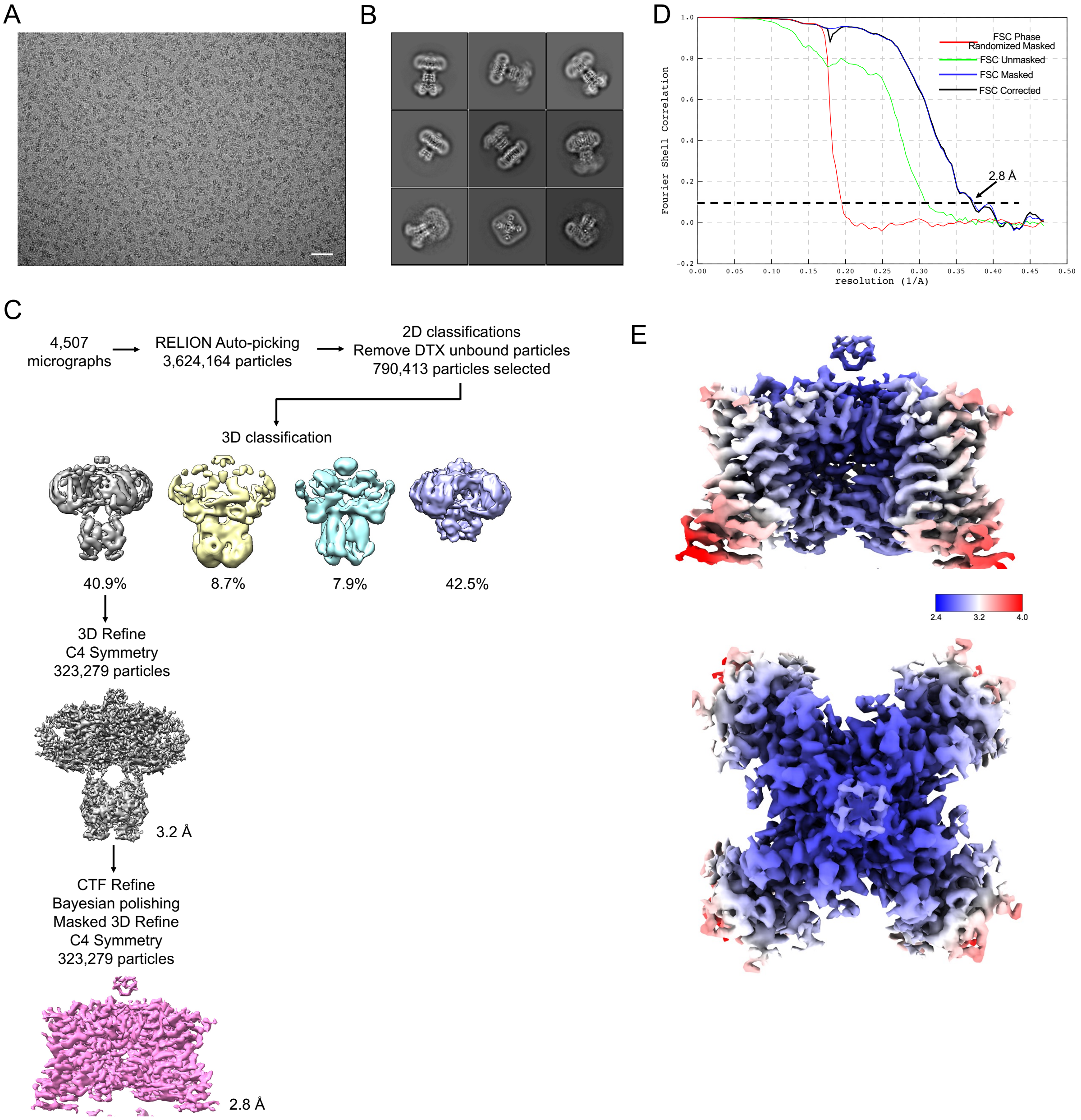


**Figure.3 - figure supplement 1 Cryo-EM of Kv1.2 DTX.** (A) Representative micrograph. (scale bar 20 nm) (C) Representative 2D classes. (C) Cryo-EM data processing workflow. (D) Gold standard FSC resolution estimation. (F) Local resolution estimation.

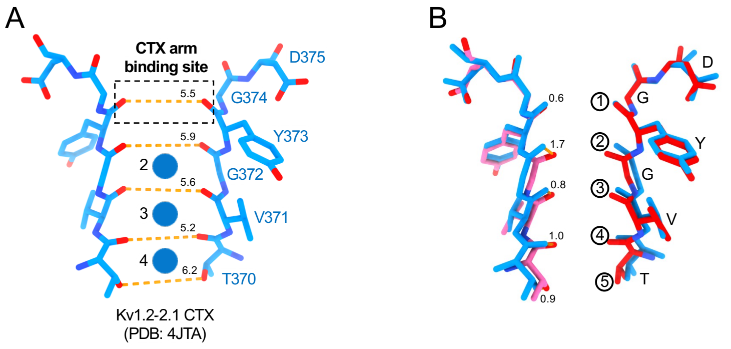


**Figure.3 - figure supplement 2 Structural comparison of Kv1.2 DTX bound structure with Kv1.2-2.1 CTX bound structure.** (A) Side view of the selectivity filter of Kv1.2-2.1 CTX bound conformation. Orange dashed lines show the distances between carbonyl oxygens, Potassium ions are shown as blue balls. (B) Superposition of Kv1.2 DTX (red) and Kv1.2-2.1 CTX (blue) selectivity filter structures. Carbonyl displacements are given in Å.

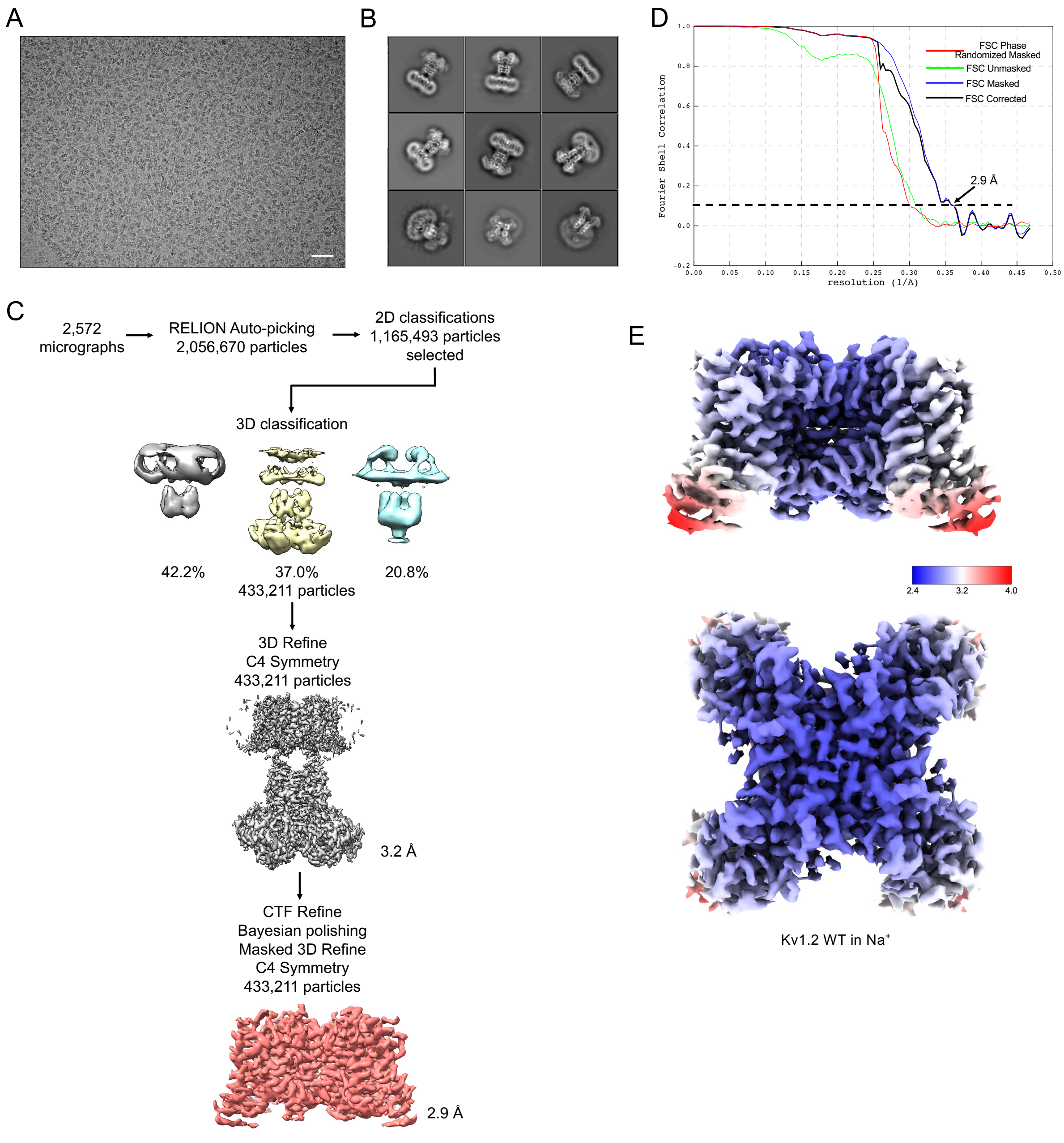


**Figure.4 - figure supplement 1 Cryo-EM of Kv1.2 in Na$^+$.** (A) Representative micrograph. (scale bar 20 nm) (C) Representative 2D classes. (C) Cryo-EM data processing workflow. (D) FSC resolution estimation. (F) Local resolution estimation.

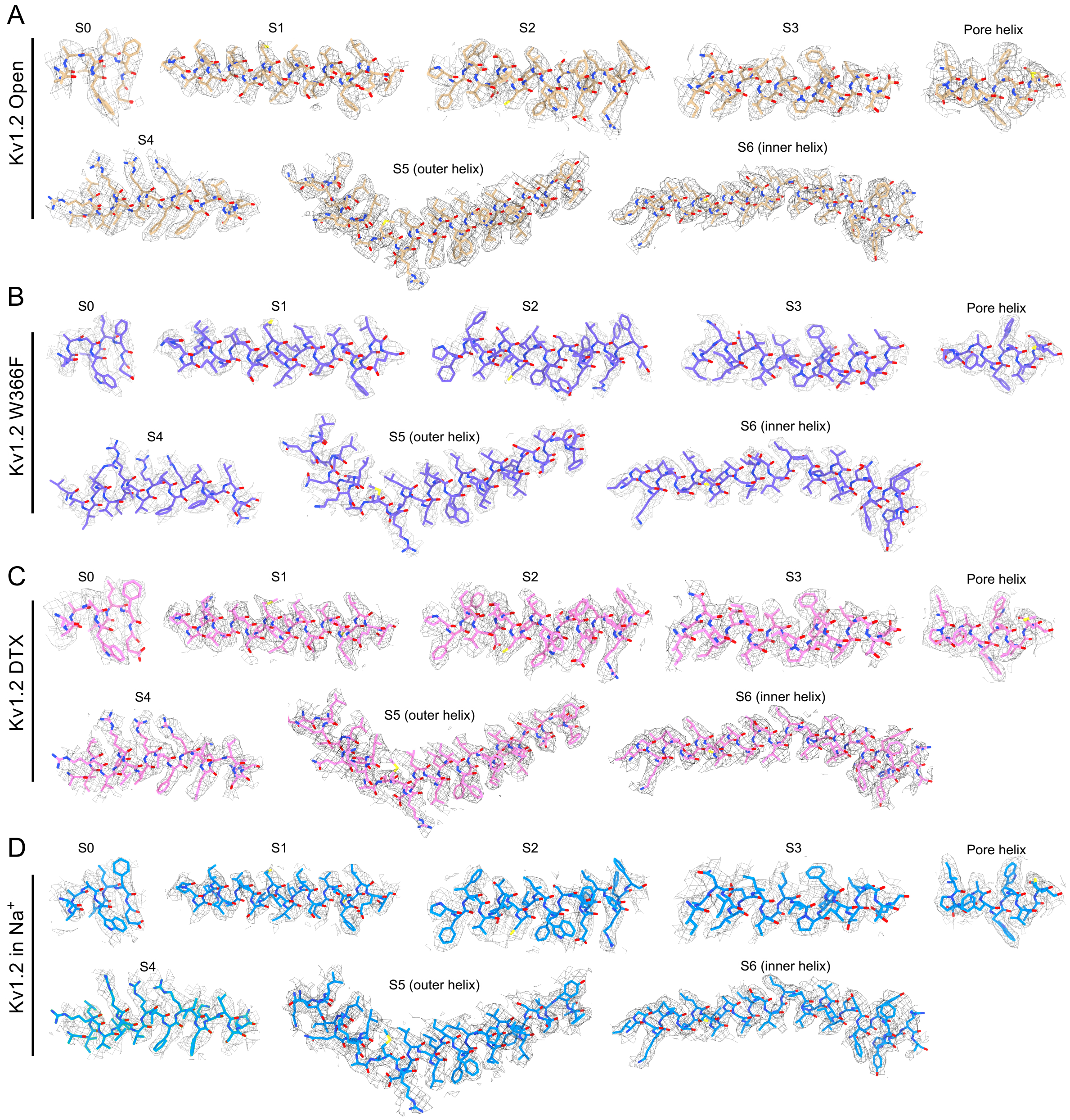


**Figure.4 - figure supplement 2 Comparisons of cryo-EM density map and model for each Kv1.2 structure reported here.**

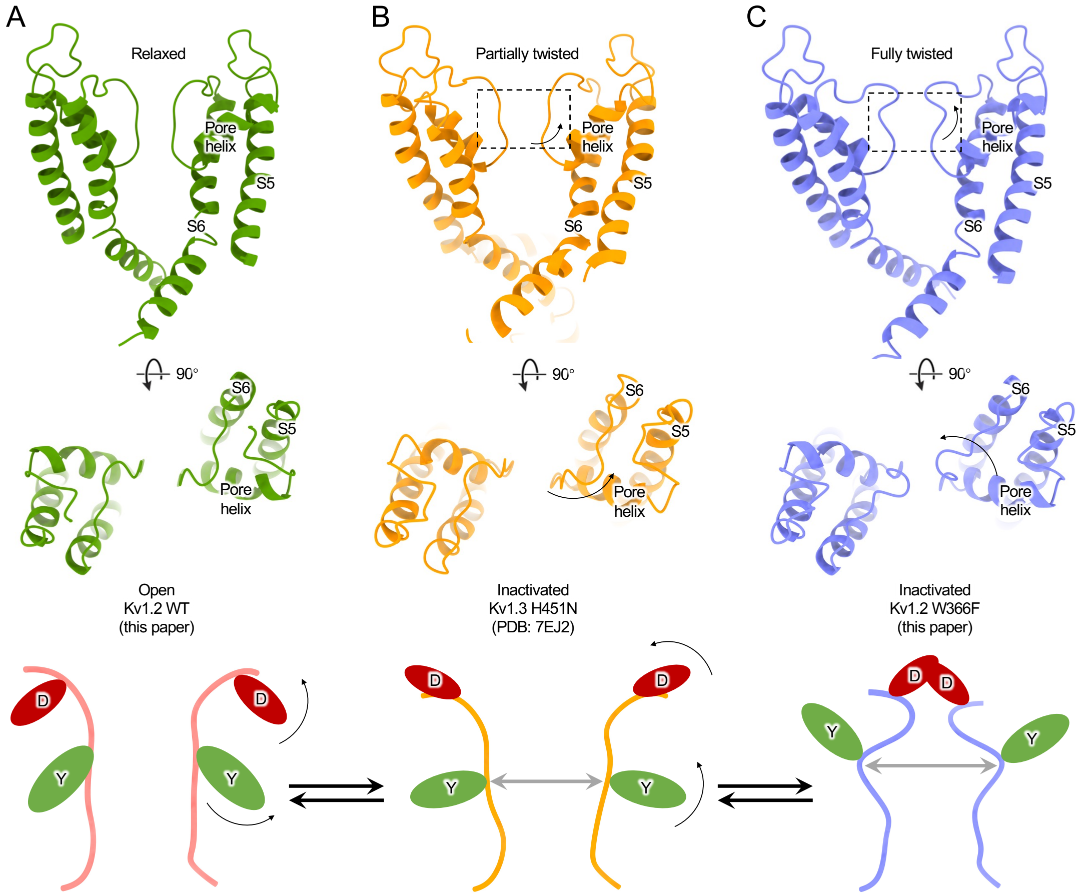


**Figure.6 - figure supplement 1 Conformational changes of Kv channel inactivation.** (A) Kv1.2 WT PD in green (B) Kv1.3 H451N PD in orange and (C) Kv1.2 W366F PD in orchid represent the relaxed, partially twisted and fully twisted P-loop respectively. Cartoon illustration of (A) relaxed, (B) partially twisted and (C) fully twisted selectivity filter P-loop of Kv channels. D30' and Y28' residue side chains are shown as red and green ovals.
